# Phylogeographic and Potential Distribution of Wild Apricot (*Prunus armeniaca*) in Xinjiang: Insights From Chloroplast/Nuclear DNA and Ecological Niche Modeling

**DOI:** 10.1002/ece3.73206

**Published:** 2026-03-09

**Authors:** Mingyu Li, Xiaolan Wu, Mengfan Cui, Tao Hu, Chenyang Ma, Chen Yuan, Chenxi Liu, Deyin Cao, Wenwen Li, Kai Jia

**Affiliations:** ^1^ College of Horticulture Xinjiang Agricultural University Urumqi China

**Keywords:** historical dynamics, phylogeography, potential distribution, *Prunus armeniaca*

## Abstract

As the wild progenitor of cultivated apricot, Xinjiang wild apricot is a key resource for ecosystem stability and germplasm conservation. We analyzed its phylogeography in the Ili wild fruit forests using two chloroplast DNA regions (*rpl32‐trnL*, *ndhC‐trnV*) and one single‐copy nuclear locus (DXH). Genetic variation was mainly within populations, with weak among‐population differentiation. cpDNA and nuclear data showed discordant spatial patterns, indicating different demographic signals from seed‐mediated versus pollen‐mediated processes. cpDNA neutrality and mismatch analyses did not support recent overall expansion and were more consistent with contraction/bottleneck dynamics, although the dominant cpDNA lineage (h4) retained a post‐LGM expansion signal (~19.6 ka). In contrast, DXH supported recent expansion, strongest in the Yining County population. Isolation‐by‐distance was significant for cpDNA but weak and nonsignificant for DXH, consistent with stronger geographic structuring of maternally inherited variation. MaxEnt models identified precipitation in the wettest season (100–135 mm), precipitation in the warmest season (90–120 mm), and soil sand content (6%–38%) as key predictors of distribution. Suitable habitat is currently concentrated in the Ili Kazakh Autonomous Prefecture, with projected expansion under future climate scenarios. These results provide an integrated view of historical demography and contemporary habitat suitability, and offer a basis for conservation planning and sustainable use of Xinjiang wild apricot genetic resources.

## Graphical abstract

This study employed integrated analyses of chloroplast DNA and nuclear genes, which revealed significant divergence in genetic structure between the two genomic compartments in Xinjiang wild apricots, reflecting complex evolutionary processes influenced by natural selection, genetic drift, and gene flow. Population history analyses indicated an absence of significant expansion in the maternal lineages, representing a potential bottleneck event, whereas nuclear data provided evidence of recent expansion, most prominently in the Yining County population. Additionally, ecological niche modeling projected an expansion of the species' suitable habitat under future climate scenarios.

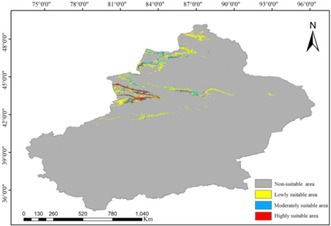

## 1 Introduction

The wild apricot ( *Prunus armeniaca* ) belonging to the genus *Prunus* within the Rosaceae family is a significant relict species of the Tertiary warm‐temperate broadleaf forests (Zhang and Zhang 2003). It was predominantly distributed in the low‐elevation mountains of the Ili River Valley in Xinjiang and can form continuous pure stands in ecologically favorable locations (Li et al. 2020). As the globally recognized wild progenitor group for cultivated apricots, the wild apricot plays a pivotal role in the history of domestication (Groppi et al. 2021). Previous studies have reported that wild apricot encompasses 44 infraspecific types with rich genetic diversity (Wang et al. 1997). As one of China's diversity hotspots for *Prunus* species, the Ili River Valley, with its distinct topographic and climatic conditions, has served as a refugium for numerous ancient species throughout geological time. Wild apricot ( *P. armeniaca* ) populations here have undergone protracted natural selection and adaptation, acquiring a unique genetic background (Li et al. 2021). This study aimed to further elucidate the phylogeographic characteristics of wild apricots in the Ili River Valley, reveal their historical distributional dynamics, and concurrently evaluate the impact of future climate change on shifts in their persistence potential; this study was critical for formulating science‐based conservation strategies for wild apricot germplasm.

Phylogeography integrates markers with varying mutation rates to reveal how past and present processes shape genetic patterns within populations. It aims to decipher the temporal and spatial composition of population structures and uncover the underlying evolutionary and ecological processes (Chen et al. 2015; Avise et al. 1987). Phylogeographic analyses of diverse plant taxa analyses have provided paradigms for understanding the evolutionary histories of species, revealing how geological events and climate change have shaped plant phylogenetic structures (Qiu et al. 2011; Xu et al. 2010; Liu et al. 2025; Chen, Xing, et al. 2012). Since species phylogenetic structures are deeply influenced by population history, researchers commonly analyze the spatial distribution of genetic lineages using markers such as chloroplast DNA (cpDNA) and nuclear genes, including single‐copy nuclear genes (SCN) and nuclear ribosomal DNA (nrDNA), to infer historical demographic processes (Witharana et al. 2025; Qin et al. 2025; Zhu et al. 2024; Zhang, Wei, et al. 2019). Previous studies using population genomics revealed that the Chinese apricot and European apricot represent two distinct gene pools independently domesticated from different wild populations in Central Asia. Those studies showed that the genome of the European apricot exhibited stronger signals of selective elimination, while the evolutionary trajectory of Chinese cultivated apricots was dominated by extensive gene flow. This resulted in a unique population characterized by high genetic diversity and a complex admixture background, indicating frequent gene exchange with local wild relatives or regional cultivars post‐domestication, which shaped its intricate genetic history (Groppi et al. 2021). A phylogeographic analysis of *Prunus sibirica* was conducted using three molecular markers—cpDNA (*atpB*‐*rbcL* and *trnQ*‐*rps16*), chloroplast simple sequence repeats (cpSSR), and nuclear simple sequence repeats (nSSR). This revealed significant spatial heterogeneity in genetic variation, the presence of multiple glacial refugia, and an evolutionary history primarily driven by geographic isolation (Wang et al. 2017). Similarly, a study on two peony species ( *Paeonia delavayi* and *Paeonia ludlowii*) from the Himalayan‐Hengduan Mountains, combining cpDNA (*trnL‐trnF*, *rps16‐trnK*, *trnH‐psbA*) and SCN markers, uncovered clear genetic and ecological niche differentiation. Their phylogenetic divergence was jointly driven by geographic isolation and environmental heterogeneity. The study also confirmed a historical population bottleneck in *P. delavayi* , supporting the role of geological and climatic changes in speciation and diversity maintenance within this clade (Zhao et al. 2021). Research on East Asian *oaks* using cpDNA has elucidated their phylogeographic history, demonstrating that chloroplast haplotype sharing among *Quercus acutissima* , *Quercus serrata* , and *Quercus variabilis* results from retained ancestral polymorphism coupled with historical gene flow, thereby shaping their current spatial genetic structure (Li, Wang, et al. 2022; Li, Zhang, et al. 2022). For North American *Parthenocissus* species, analyses of cpDNA (*trnL‐F*, *rps16*, and *trnC‐petN*) and nrDNA provided the first systematic insight into the evolutionary dynamics of this temperate vine. The analyses revealed that a northward decrease in genetic diversity was observed, suggesting likely glacial refugia and postglacial northward expansion via long‐distance dispersal and gene flow (Médail and Diadema 2009). Analysis of cpDNA (*psbA‐trnH*, *trnD‐psbM*) and nrDNA in *Morella nana*, endemic to the Yunnan‐Guizhou Plateau, confirmed genetic divergence between eastern and western lineages separated by the Wumeng Mountains. This finding underscored the role of this mountain range in geographic isolation and provides key evidence for at least two Quaternary glacial refugia in the region (Wu et al. 2024). The evolutionary history of *Thuja*, a Tertiary relict genus with a disjunct distribution between East Asia and North America, was investigated using SCN and cpDNA genomes. The study revealed discordant topologies between nuclear and plastid phylogenetic trees, which were primarily attributed to incomplete lineage sorting, which was potentially linked to rapid Miocene diversification and large effective population sizes in ancestral lineages (Li et al. 2022). In summary, phylogeography reveals the historical causes and geographical constraints underlying plant population dynamics across space and time. These insights not only deepen our understanding of species formation and adaptation but also provide a critical foundation for the conservation and utilization of plant genetic resources.

Ecological niche models (ENMs) are computational models used in ecology and conservation biology to predict species' potential distributions and habitat suitability. They utilize species distribution data alongside environmental variables such as temperature, precipitation, and soil characteristics to identify factors influencing species distribution patterns. Among these, the Maximum Entropy (MaxEnt) model is currently the most widely applied ecological niche model (Feng et al. 2024; Ye et al. 2025). By predicting the potential distribution ranges of different species under varying climatic conditions and examining shifts in suitable habitats, MaxEnt provides a theoretical basis for plant germplasm resource conservation (Chaurasia et al. 2024; Shi et al. 2024). Moraira et al. (2020) used MaxEnt modeling to predict that the future distribution range of the invasive species *Kalanchoe* × *houghtonii* could shrink by up to one‐third of its current range, suggesting a link to its Crassulacean acid metabolism pathway. To assess the impacts of climate change and human activities on the introduction adaptability of introduced southern magnolia ( *Magnolia grandiflora* ), geographic distribution data from its North American native range and Chinese introduction sites were used to predict potential distributions under future climate scenarios. Results indicated that the southern magnolia would expand northwestward and northeastward in both regions while its southern suitable habitat would shrink. These findings provide guidance for conservation, introduction planning, and sustainable utilization strategies for this species (Zhang, Wang, et al. 2024). The MaxEnt model, with its ability to quantify key environmental factors and predict distribution dynamics, has proven effective for forecasting suitable ranges of rare, endangered, and economically valuable species (Zhang, Chase, and Liao 2024; Zhang, Nizamani, et al. 2024; Zhang, Wang, et al. 2024; Xue et al. 2024).

This study analyzed the genetic diversity of wild apricot ( *P. armeniaca* ) populations in the Ili River Valley, Xinjiang, using cpDNA (*rpl32‐trnL* and *ndhC‐trnV*) and SCN (*DXH*) markers. It elucidated the population genetic structure, traced the phylogenetic differentiation history, and investigated whether the populations experienced population expansion or contraction events. Additionally, environmental variable analysis was conducted using the MaxEnt model to simulate and predict changes in potential suitable habitats under current and future scenarios. These findings provide a robust theoretical basis for developing conservation strategies for this rare germplasm resource.

## 2 Materials and Methods

### 2.1 Sample Collection

Field surveys were conducted, resulting in the collection of a total of 201 samples from 12 populations. The sampling strategy was as follows: at least 10 individuals were selected from each population (if fewer than 10 individuals were present, all observed individuals were collected) spaced at intervals of at least 50 m. The latitude, longitude, and elevation of each population were recorded. Five to ten fresh, pest‐free young leaves were collected from each individual, dried in silica gel, and preserved. Detailed information on sample collection is presented in Table 1.

**TABLE 1:** Sample locations and sample size of *Prunus armeniaca* .

Population code		Sampling location	Number of samples	Latitude (N)	Longitude (E)	Altitude (m)
Xinyuan County population	XHG	Xinjiang Uygur Autonomous Region	20	43°32′16.44″	83°25′57.35″	1143
	ZWY	Xinjiang Uygur Autonomous Region	15	43°22′42.24″	83°36′26.60″	1427
Gongliu County population	BL	Xinjiang Uygur Autonomous Region	15	43°15′0.10″	82°51′1.47″	1305
	DMH	Xinjiang Uygur Autonomous Region	18	43°14′2.30″	82°44′24.83″	1250
	XMH	Xinjiang Uygur Autonomous Region	16	43°13′28.31″	82°43′18.53″	1231
	YLGD	Xinjiang Uygur Autonomous Region	18	43°22′35.55″	82°7′18.44″	1139
Yining County population	AWZ	Xinjiang Uygur Autonomous Region	16	44°7′38.47″	81°38′38.54″	1155
	PLQG	Xinjiang Uygur Autonomous Region	15	44°9′11.67″	81°31′11.56″	1150
Huocheng County population	XXG	Xinjiang Uygur Autonomous Region	15	44°26′10.09″	80°49′50.76″	1217
	MZD	Xinjiang Uygur Autonomous Region	16	44°26′26.60″	80°47′25.07″	1184
	MJT	Xinjiang Uygur Autonomous Region	17	44°25′39.87″	80°46′52.88″	1118
	MZG	Xinjiang Uygur Autonomous Region	20	44°24′6.37″	80°42′49.02″	1222

### 2.2 DNA Extraction, PCR Amplification and Sequencing

Total DNA was extracted from leaf tissue using a plant genomic DNA extraction kit (TIANGEN, Beijing, China). Based on the previously assembled wild apricot chloroplast genome data from our research group, five polymorphic cpDNA fragments were preliminarily screened through multiple sequence alignment, and specific primers were designed. Concurrently, 10 single‐copy nuclear genes were pre‐selected based on previous reports (Hu et al. 2021). Following validation of PCR amplification efficiency and sequencing performance, two cpDNA fragments (*rpl32‐trnL* and *ndhC‐trnV*) and one single‐copy nuclear locus (*DXH*) were selected for subsequent analyses. This marker combination was designed to capture complementary inheritance signals: cpDNA markers were used to infer maternal lineage history and seed‐mediated dispersal, whereas *DXH* was used to represent biparental variation influenced by both seed and pollen flow. Candidate loci were retained only when they showed stable amplification, reliable bidirectional sequencing, and sufficient polymorphism across populations. Although a single nuclear locus cannot fully represent genome‐wide demographic history, this cpDNA‐nuclear framework has been widely used for broad‐scale phylogeographic inference when whole‐genome data are not yet available. Primer information was detailed in Table S1. The PCR reaction volume totaled 25 μL, comprising 12.5 μL of 2× Taq PCR Master Mix, 1 μL each of forward and reverse primers, 1 μL of genomic DNA template, and 9.5 μL of ddH₂O. The amplified products were purified and then sent to Sangon Biotech (Shanghai) Co. Ltd. for bidirectional Sanger sequencing and sequence assembly to ensure the reliability of the sequence information.

### 2.3 Data Analysis

#### 2.3.1 Sequencing Result Processing

The sequencing chromatograms were imported into Chromas (Technelysium Pty Ltd., https://technelysium.com.au/wp/Chromas) to visualize peak patterns and perform manual inspection. For base calling, distinct strategies were employed for the nuclear and chloroplast datasets. For the nuclear *DXH* gene, heterozygous sites were identified by examining double peaks in the chromatograms and confirmed heterozygous positions were annotated using IUPAC degenerate base codes. The gametic phase was subsequently inferred using the PHASE algorithm (Stephens et al. 2001). For the cpDNA dataset, sequences were directly generated and stored in FASTA format as heterozygous sites are theoretically absent due to the haploid nature of the chloroplast genome. Finally, for all datasets, multiple sequence alignment, manual refinement, and primer region trimming were conducted using MEGA v.7.0 (Li et al. 2023; Wu et al. 2024). The final analytical dataset comprised the combined *rpl32‐trnL* and *ndhC‐trnV* chloroplast sequences, as well as the *DXH* nuclear gene.

#### 2.3.2 Analysis of Population Genetic Diversity and Haplotype Phylogeny

DnaSP v.5.0 software was used to screen and detect haplotypes, and to calculate haplotype diversity (*H*d), nucleotide diversity (*P*i), and genotypic polymorphism. A haplotype network map was visualized using Popart v.1.7, and haplotype geographic distribution maps were constructed with ArcGIS v.10.8 software (Wu et al. 2024). Analysis of molecular variance (AMOVA) in Arleequin v.3.5 was employed to calculate the interpopulation genetic differentiation index: the fixation index (*F*st) (Yi et al. 2020). To assess the isolation‐by‐distance (IBD) pattern, we linearized pairwise *F*st values using the transformation *F*st/(1 − *F*st). Geographical distances were calculated using the latitude and longitude coordinates of each population and were log_10_‐transformed. A Mantel test (Pearson correlation coefficient with 9999 permutations) was then performed in R using the vegan package to examine the correlation between genetic and geographical distances.

#### 2.3.3 Dynamic Analysis of Population History

The neutrality tests were employed to analyze the population history dynamics of wild apricot, with inferences based on Tajima's *D* and Fu's *Fs* values. Significantly negative values for both statistics (*p* < 0.05) indicated that the wild apricot population had recently undergone a rapid expansion event; conversely, they suggested that the population had experienced a contraction event or bottleneck. Arlequin v.3.1.1 software was used to perform Tajima's *D* and Fu's *Fs* tests to assess whether wild apricot populations experienced expansion or bottleneck effects. DnaSP v.6 software was employed for mismatch distribution analysis between and within wild apricot populations (He et al. 2025).

#### 2.3.4 Present and Past Ecological Niche Modeling

The environmental data selected for this study encompassed 19 climate factors and 3 topographic factors under the SSP126 and SSP585 climate scenarios for the contemporary period (1970–2000) and two future periods (2061–2080 and 2080–2100). All data were sourced from the World Climate Database (http://www.worldclim.org/), with a 30‐arc‐second resolution. Future climate factors were modeled using the Beijing Climate Center Climate System Model version 2 with Medium Resolution (BCC‐CSM2‐MR) (Fick and Hijmans 2017). Soil variables were derived from eight soil factors within the 0–20 cm shallow soil layer data of the World Soil Coordinated Database Version 2.0 from the Food and Agriculture Organization of the United Nations (https://www.fao.org/). ArcGIS v.10.8 was used to process raster basemaps for all environmental variables.

Nineteen climatic factors, three topographic factors, eight soil factors, and species distribution points were imported into the MaxEnt model for preliminary analysis operation to obtain the contribution rate and replacement importance of each environmental variable. Subsequently, correlation analysis and a multicollinearity test were conducted in the R environment. A Pearson correlation matrix was calculated using 20,000 pixels randomly sampled from the environmental layers at a uniform resolution. If |*r*| ≥ 0.8, the variable with stronger ecological relevance and a higher contribution to the MaxEnt model (Dormann et al. 2013) was retained, and the correlated variable was excluded. Variance inflation factor (VIF) analysis was then performed, and variables with VIF ≥ 10 were iteratively removed until all remaining variables had a VIF < 10 (Wei et al. 2018). Finally, seven relatively independent and important environmental variables were screened, including mean annual air temperature (Bio1), precipitation of the wettest quarter (Bio16), precipitation of the warmest quarter (Bio18), mean temperature of the wettest quarter (Bio8), altitude (Ele), sand content (Sand), and slope (Slo).

The distribution records of wild apricot were primarily obtained from the Chinese Virtual Herbarium (CVH) and field surveys conducted in this study, totaling 245 occurrence points (44 from CVH and 201 from field surveys). To mitigate model overfitting resulting from sampling bias and spatial autocorrelation, spatial rarefaction was performed on the data using ArcGIS v.10.8. Using the grid resolution of the environmental variables (1 km × 1 km) as the criterion, only one occurrence point was retained per grid cell. Ultimately, 29 spatially independent occurrence points were selected for ecological niche modeling. To ensure the robustness of the prediction results, the MaxEnt model was implemented in conjunction with the ENMeval package for parameter optimization (Muscarella et al. 2014). Four feature class (FC) types (L, LQ, H, LQH) were tested, and the regularization multiplier (RM) was tuned from 0.5 to 4.0 in increments of 0.5. Based on 4‐fold cross‐validation, the optimal parameter combination was selected by minimizing the corrected Akaike Information Criterion (AICc) (Warren and Seifert 2011). Using the selected optimal parameters, the MaxEnt model was run with the 29 occurrence points randomly partitioned into a 75% training set and a 25% test set (Qin et al. 2017). The model was executed 10 times, and the average output was taken as the final prediction. Model performance was evaluated using the area under the receiver operating characteristic curve (AUC) (Chang et al. 2021). Preliminary results showed that the mean test AUC was 0.987 (SD = 0.004), significantly exceeding the accuracy threshold of 0.9, which indicated that the parameter‐optimized model exhibited high predictive reliability under the present sample size. Variable importance analysis revealed that annual mean temperature (Bio1), precipitation of the wettest quarter (Bio16), and elevation (Ele) were the dominant factors influencing the distribution of wild apricot.

The average prediction results of the MaxEnt model were exported to ASCII format and converted into raster data in ArcGIS v.10.8. The prediction probability was divided into four categories: non‐suitable area, low suitable area, medium suitable area, and high suitable area using the Jenks natural breaks method. The areas of each suitable area category under different greenhouse gas emission scenarios and time periods were calculated, and spatial distribution maps were created to analyze the potential distribution patterns and trends of wild apricot under future climate change (Yousefzadeh et al. 2022).

## 3 Result

### 3.1 Population Genetic Diversity and Haplotype Distribution

**FIGURE 1:**
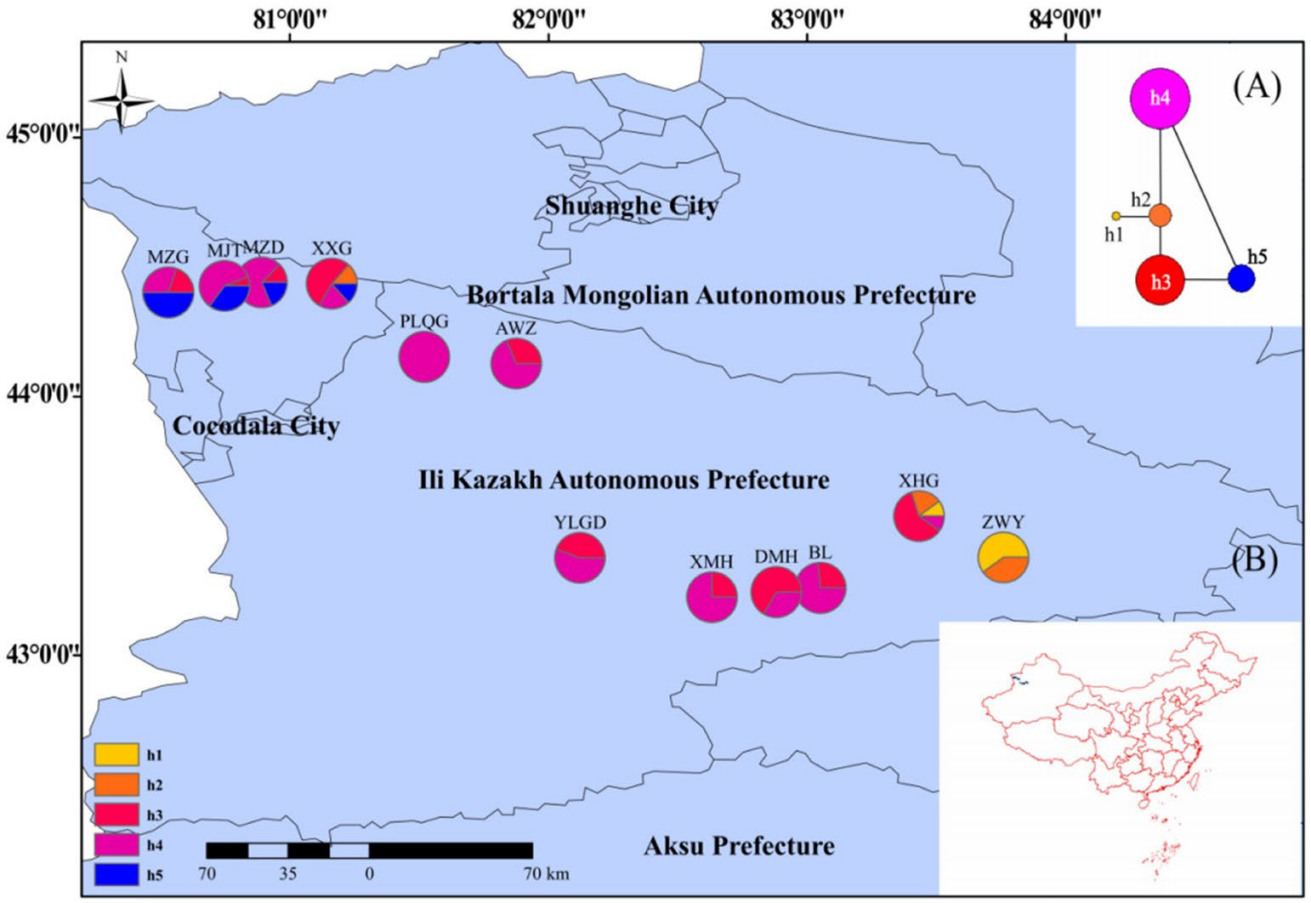
Haplotype network (A) and geographical distribution of chloroplast haplotypes (B) for *Prunus armeniaca* , h1–h5 represent haplotypes 1–5, respectively, and the pie chart indicates the frequency of haplotypes in each population.

Based on cpDNA sequences, five haplotypes were identified (Table 2). Wild apricot exhibited moderate genetic diversity in the chloroplast genome (*H*d = 0.646, *P*i = 0.99 × 10^−3^). Haplotype h4, which showed the widest distribution and highest frequency across populations, occupied the central position in the network and connected most low‐frequency haplotypes (Figure 1A), forming a star‐like pattern. This pattern suggests a lineage‐level signal of demographic expansion associated with the dominant h4 lineage, rather than direct evidence of population‐wide expansion across all maternal lineages. Based on mismatch analysis for the h4‐centered lineage pattern, we used *τ* = 0.07 to estimate expansion timing. Using a chloroplast mutation rate of 1.5 × 10^−9^, the estimated time was approximately 19.6 ka (Table S2). Therefore, this signal was more consistent with a post‐LGM (late‐glacial/deglacial) expansion event. Although the origin of h4 may predate the LGM, its increase in frequency and the diversification of derived haplotypes likely occurred after the LGM. In contrast, endemic haplotypes (e.g., h1 in the XHG population) exhibited stronger local differentiation, suggesting that some populations have developed unique genetic structures and accumulated new mutations under geographical isolation. The haplotype geographic distribution map (Figure 1B) further revealed significant spatial heterogeneity in haplotype composition: the XHG and XXG populations exhibited the highest haplotype richness (4 haplotypes each), whereas the PLQG population showed complete haplotype fixation (only haplotype h4). Significant genetic differentiation was observed among populations (Table 2), with populations such as XXG (*H*d = 0.686) and MZG (*H*d = 0.653) maintaining relatively high diversity. Notably, haplotype h5 was exclusively concentrated in Huocheng County populations (XXG, MZD, MJT, and MZG), exhibiting distinct phylogeographic patterns. This distribution pattern suggests that wild apricot populations may have undergone genetic differentiation driven by geographic isolation.

**TABLE 2:** Genetic diversity parameters and haplotype numbers in *Prunus armeniaca* populations based on cpDNA.

Population code	*H*d	Nucleotide polymorphism	Haplotype distribution
		*P*i × 10^−3^	
ZWY	0.514	0.520	h2(9)h3(6)
XHG	0.611	0.700	h1(2)h2(4)h3(12)h4(2)
BL	0.419	0.840	h3(4)h4(11)
DMH	0.471	0.950	h3(12)h4(6)
XMH	0.400	0.750	h3(4)h4(12)
YLGD	0.523	1.050	h3(8)h4(10)
AWZ	0.458	0.920	h3(5)h4(11)
PLQG	0.000	0.000	h4(15)
XXG	0.686	0.960	h2(2)h3(8)h4(3)h5(2)
MZD	0.508	0.700	h3(2)h4(11)h5(3)
MJT	0.559	0.640	h3(1)h4(10)h5(6)
MZG	0.653	0.780	h3(4)h4(6)h5(10)
Average	0.483	0.730	
All	0.646	0.990	

**FIGURE 2:**
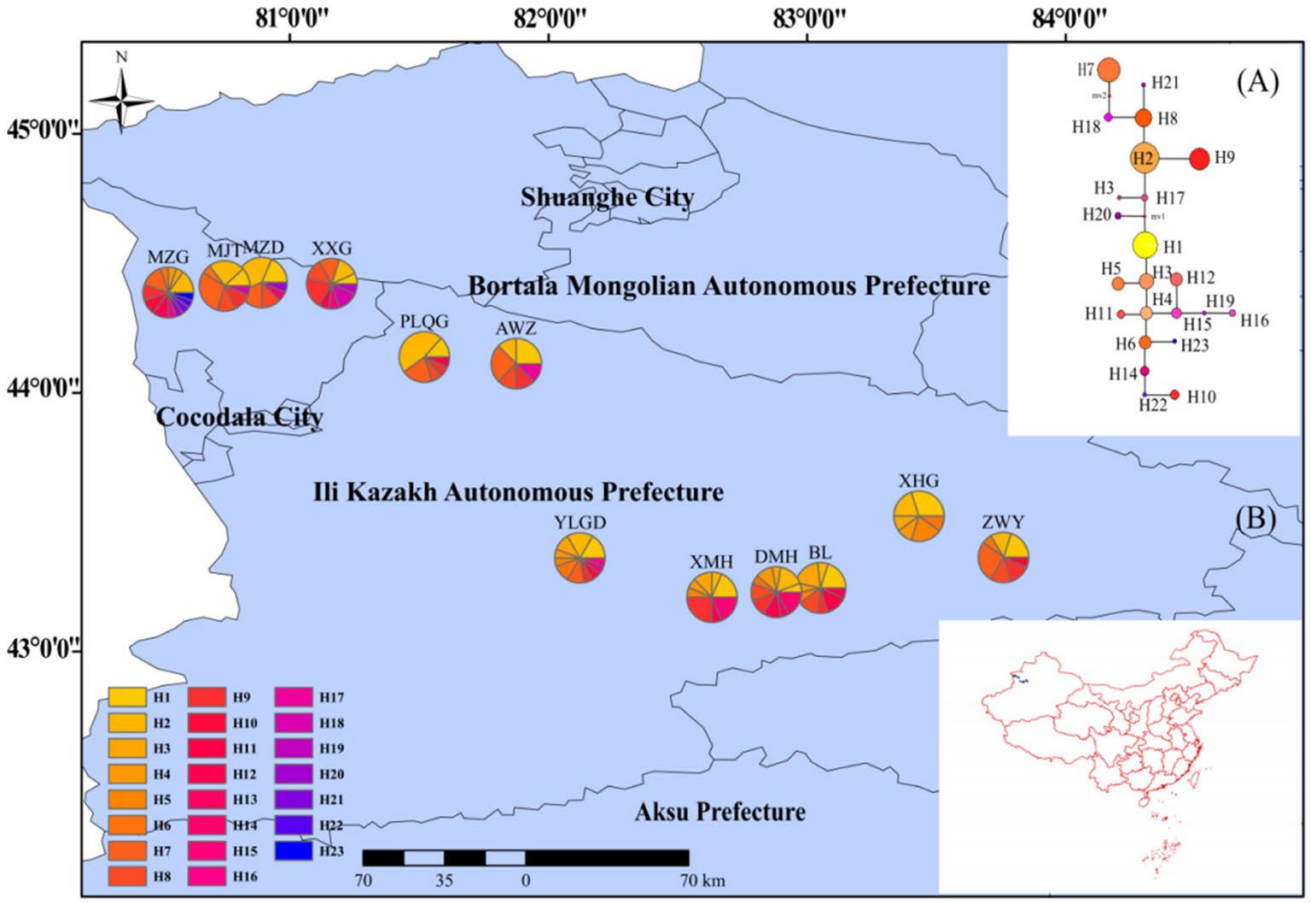
Genotype network (A) and geographical distribution (B) of *SCN* genotype for *Prunus armeniaca* . H1‐h23 represent genotypes 1–23, respectively, and the pie chart indicates the frequency of genotypes in each population.

A total of 23 genotypes were identified based on the *DXH* gene (Table 3), indicating high genetic diversity at the nuclear gene level (*H*d = 0.902, *P*i = 5.36 × 10^−3^). The genotype network map (Figure 2A) showed H2 as the dominant genotype, distributed across 38 individuals, while H13, H19, H21, H22, and H23 were rare genotypes, each present in only one individual. Significant differences in genetic diversity were observed among populations (Table 3). The MZG population exhibited not only the highest number of haplotypes (13) but also the greatest diversity (*H*d = 0.953, *P*i = 6.51 × 10^−3^); the YLGD population ranked second (11 genotypes, *H*d = 0.941); while the PLQG population retained 6 haplotypes, its nucleotide diversity was significantly reduced (*P*i = 1.98 × 10^−3^), suggesting this population may have undergone strong genetic drift effects. The genotype geographic distribution map (Figure 2B) further revealed distinct spatial genetic structures: unique haplotypes were unevenly distributed across multiple populations, including DMH (H13, H14), AWZ (H17), XXG (H18, H19), and MZG (H21, H22, H23). This distribution pattern indicated the potential existence of multiple distinct evolutionary units within wild apricot populations in the Ili River Valley, providing crucial scientific evidence for germplasm resource conservation.

**TABLE 3:** Genetic diversity parameters and genotype numbers in *Prunus armeniaca* populations based on the *DXH* locus.

Population code	*H*d	Nucleotide polymorphism	Genotype distribution
		*P*i × 10^−3^	
ZWY	0.886	3.430	H1(3)H2(2)H6(1)H7(4)H8(2)H9(2)H10(1)
XHG	0.842	6.230	H1(6)H2(4)H3(2)H4(2)H5(4)H6(2)
BL	0.924	6.420	H1(3)H2(1)H3(3)H4(1)H5(1)H8(2)H9(1)H11(2)H12(1)
DMH	0.935	6.410	H1(1)H2(3)H3(1)H4(2)H6(1)H8(2)H9(2)H12(2)H13(1)H14(3)
XMH	0.892	6.390	H1(3)H2(1)H3(2)H4(1)H5(1)H9(4)H12(1)H15(3)
YLGD	0.941	6.500	H1(3)H2(3)H3(2)H4(1)H5(1)H6(2)H7(2)H9(1)H10(1)H15(1)H16(1)
AWZ	0.867	6.250	H1(4)H2(2)H7(4)H8(2)H9(2)H17(2)
PLQG	0.762	1.980	H1(2)H2(7)H7(3)H8(1)H9(1)H11(1)
XXG	0.933	4.310	H1(1)H2(2)H7(2)H8(2)H9(3)H12(1)H15(1)H18(2)H19(1)
MZD	0.817	1.950	H1(3)H2(6)H7(3)H8(2)H14(1)H20(1)
MJT	0.860	1.880	H1(2)H2(4)H6(1)H7(5)H8(2)H9(2)H18(1)
MZG	0.953	6.510	H2(3)H3(1)H4(1)H6(1)H7(3)H9(2)H10(2)H12(2)H16(1)H20(1)H21(1)H22(1)H23(1)
Average	0.884	4.860	
All	0.902	5.360	

### 3.2 Population Genetic Differentiation

The *F*st values for the wild apricot populations based on cpDNA and SCN sequences were 0.206 and 0.150, respectively. Both fell within the range of 0.15–0.25, indicating moderate genetic differentiation among populations (Table 4). Analysis of molecular variance (AMOVA) revealed that for cpDNA sequences, among‐population variation accounted for 20.60% of the total variation, while within‐population variation constituted 79.40%. For the SCN marker, variation among populations was 14.46%, with 85.54% residing within populations. Both analyses indicated that within‐population variation was the primary source of genetic variation in wild apricot. The IBD pattern was consistent with the differentiation described above (Table 5). Linearized *F*st values for cpDNA showed a clear increase with geographical distance. Mantel tests revealed a weak but significant positive correlation between cpDNA genetic and geographical distances (Mantel's *r* = 0.238, *p* = 0.045), indicating significant spatial genetic structure at this maternally inherited marker. This suggested greater cpDNA differentiation among more distant populations. In contrast, the nuclear SCN marker showed only a nonsignificant positive trend (Mantel's *r* = 0.205, *p* = 0.073), and the IBD signal did not reach statistical significance.

**TABLE 4:** AMOVA based on cpDNA and SCN markers.

Source of variation	df	Sum of squares	Variant components	Percentage of variation	Fixation index (*F*st)
cpDNA					
Among populations	3	201.284	1.28740 Va	20.60%	
Within populations	197	977.532	4.96209 Vb	79.40%	0.206
Total	200	1178.816	6.249		
SCN					
Among populations	3	325.155	2.00046 Va	14.46%	
Within populations	197	2332.204	11.83860 Vb		
Total	200	2657.538	13.83906	85.54%	0.150

**TABLE 5:** Mantel test of correlation between genetic and geographic distances among *Prunus armeniaca* population.

Dataset	Correlation coefficient (*r*)	*p*	Permutations	Method
cpDNA	0.238	0.045*	999	Pearson
SCN	0.205	0.073	999	Pearson

* *P* 〈 0.05.

### 3.3 Population History Dynamics

**FIGURE 3:**
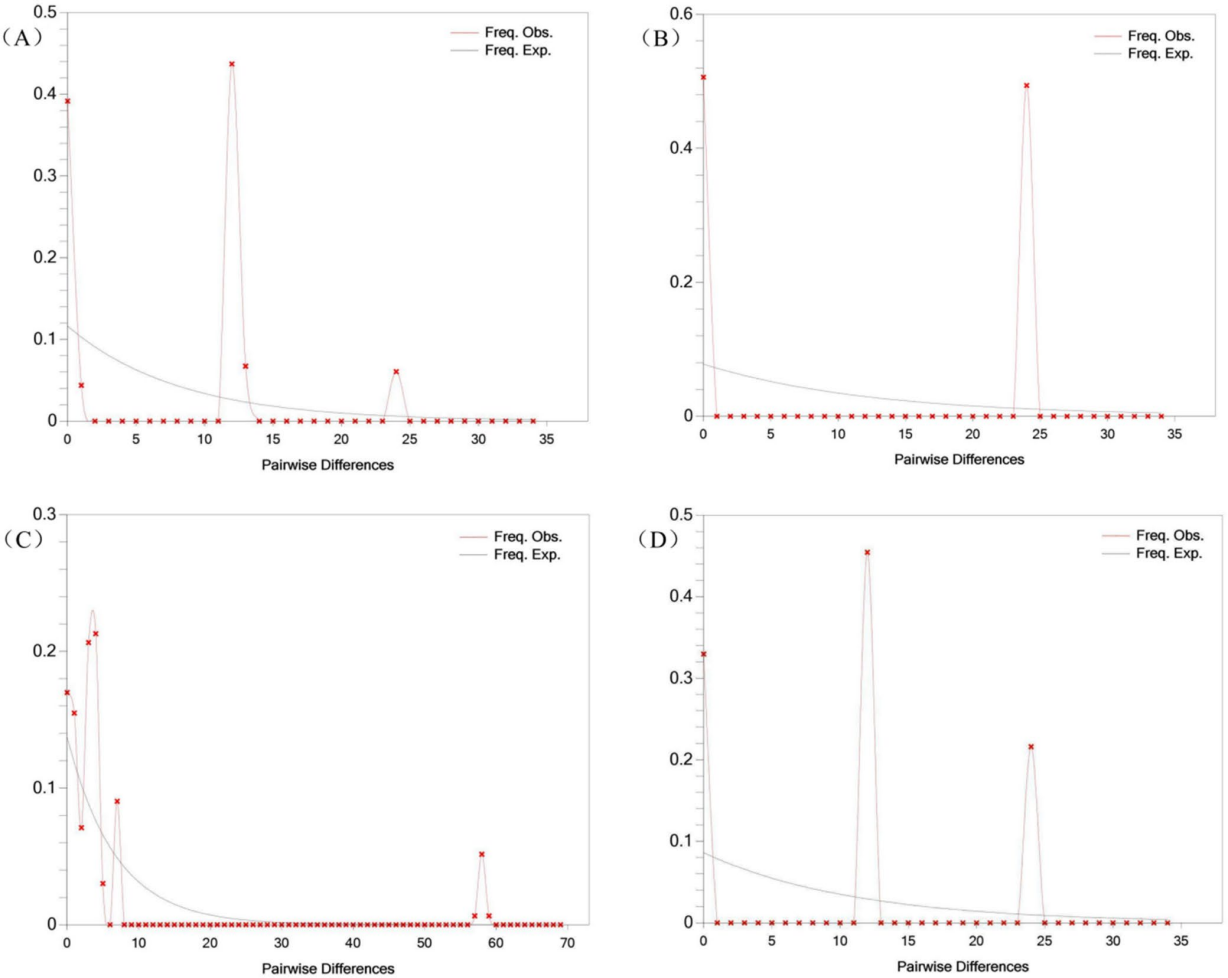
Mismatch distribution analysis based on cpDNA sequences for different populations of *Prunus armeniaca*. (E) Xinyuan County population mismatch analysis; (F) Gongliu County population mismatch analysis; (G) Yining County population mismatch analysis; (H) Huocheng County population mismatch analysis.

**FIGURE 4:**
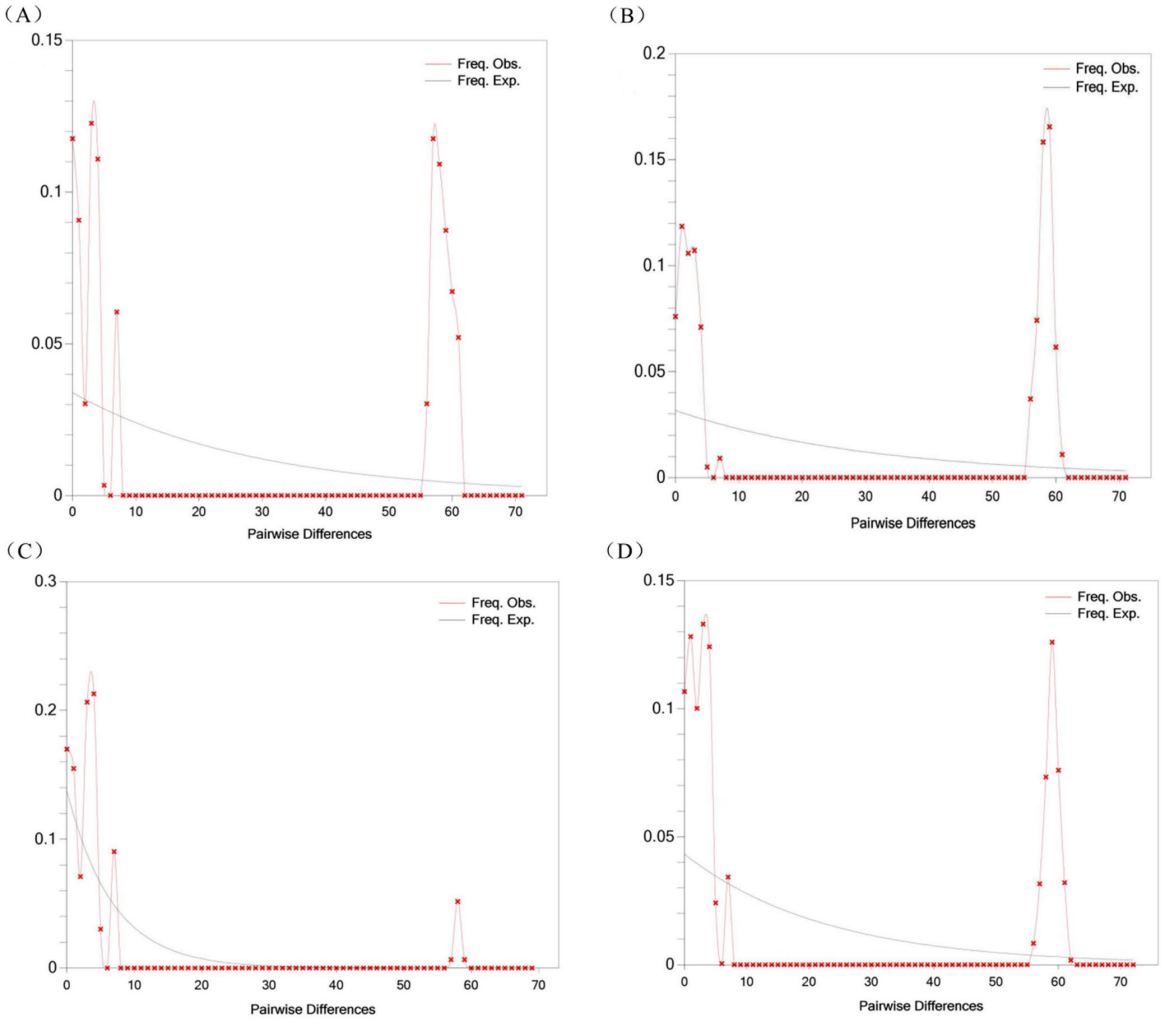
Mismatch distribution analysis based on SCN data for different populations of *Prunus armeniaca* . (A) Xinyuan County population mismatch analysis; (B) Gongliu County population mismatch analysis; (C) Yining County population mismatch analysis; (D) Huocheng County population mismatch analysis.

Based on analyses of cpDNA and SCN, wild apricot populations may have undergone distinct population dynamics throughout their evolutionary history (Table 6). cpDNA analysis revealed that both Tajima's *D* and Fu's *Fs* were positive and predominantly significant, with mismatch distributions exhibiting irregular multimodal patterns that markedly deviated from population expansion models (Figure 3). Tests of *SSD* and raggedness indices also significantly rejected expansion models (Table S3). These findings collectively indicate that wild apricot maternal lineages did not undergo significant historical expansions, but might have experienced a shared population contraction (i.e., a bottleneck effect). Importantly, this population‐level cpDNA inference does not contradict the h4‐centered lineage‐level expansion signal, because they refer to different analytical levels and potentially different temporal phases. Conversely, SCN analysis supported recent expansion events within wild apricot populations, albeit with inter‐population variation: the Yining County population exhibited a significantly negative Tajima's *D* value consistent with an expansion model; while the neutrality test results for the remaining three populations yielded significantly positive values, suggesting possible contraction. However, their mismatch distributions did not reject the expansion model (Figure 4). Collectively, SCN results broadly supported a recent expansion event in wild apricot, with the Yining County population exhibiting the most unequivocal expansion signal, indicating that this event left an imprint in its nuclear genome.

**TABLE 6:** Neutrality test statistics for *Prunus armeniaca* populations.

Population	Tajima's *D*	Fu's *Fs*
cpDNA		
Xinyuan County population	0.991 (0.794)	11.790 (1.000)
Gongliu County population	4.240 (0.998)	31.131 (1.000)
Yining County population	0.412 (0.627)	15.676 (1.000)
Huocheng County population	3.497 (0.997)	21.841 (1.000)
SCN		
Xinyuan County population	3.281 (0.994)	15.531 (1.000)
Gongliu County population	4.573 (1.000)	18.070 (1.000)
Yining County population	−2.199 (0.001)	4.297 (0.948)
Huocheng County population	2.302 (0.9400)	8.521 (0.999)

### 3.4 Analysis of Environment Variables

**FIGURE 5:**
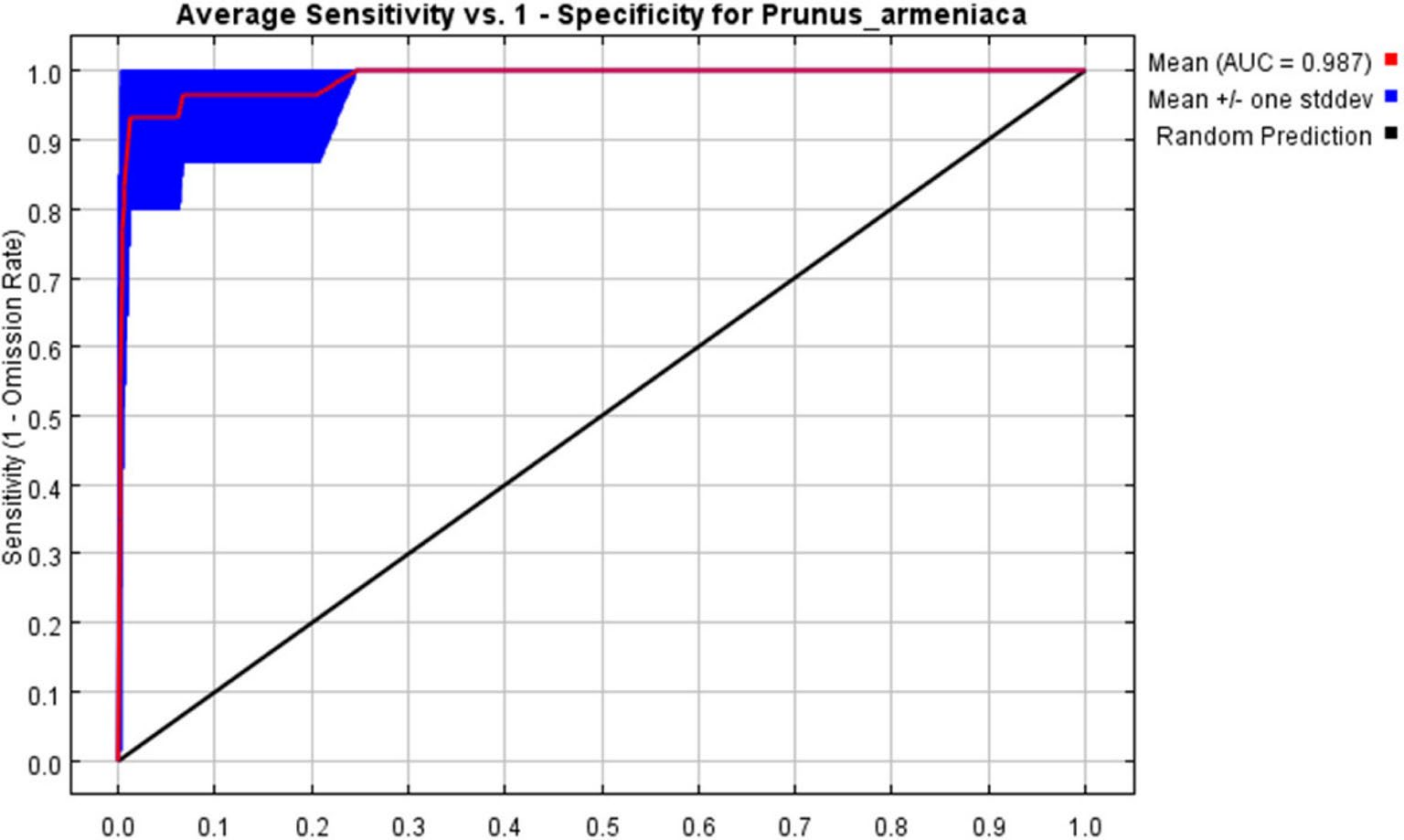
Receiver operating characteristic curve and area under the curve values for the MaxEnt model.

**FIGURE 6:**
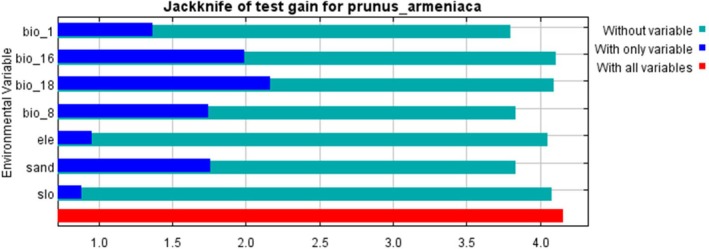
Jacknife test of variable importance.

**FIGURE 7:**
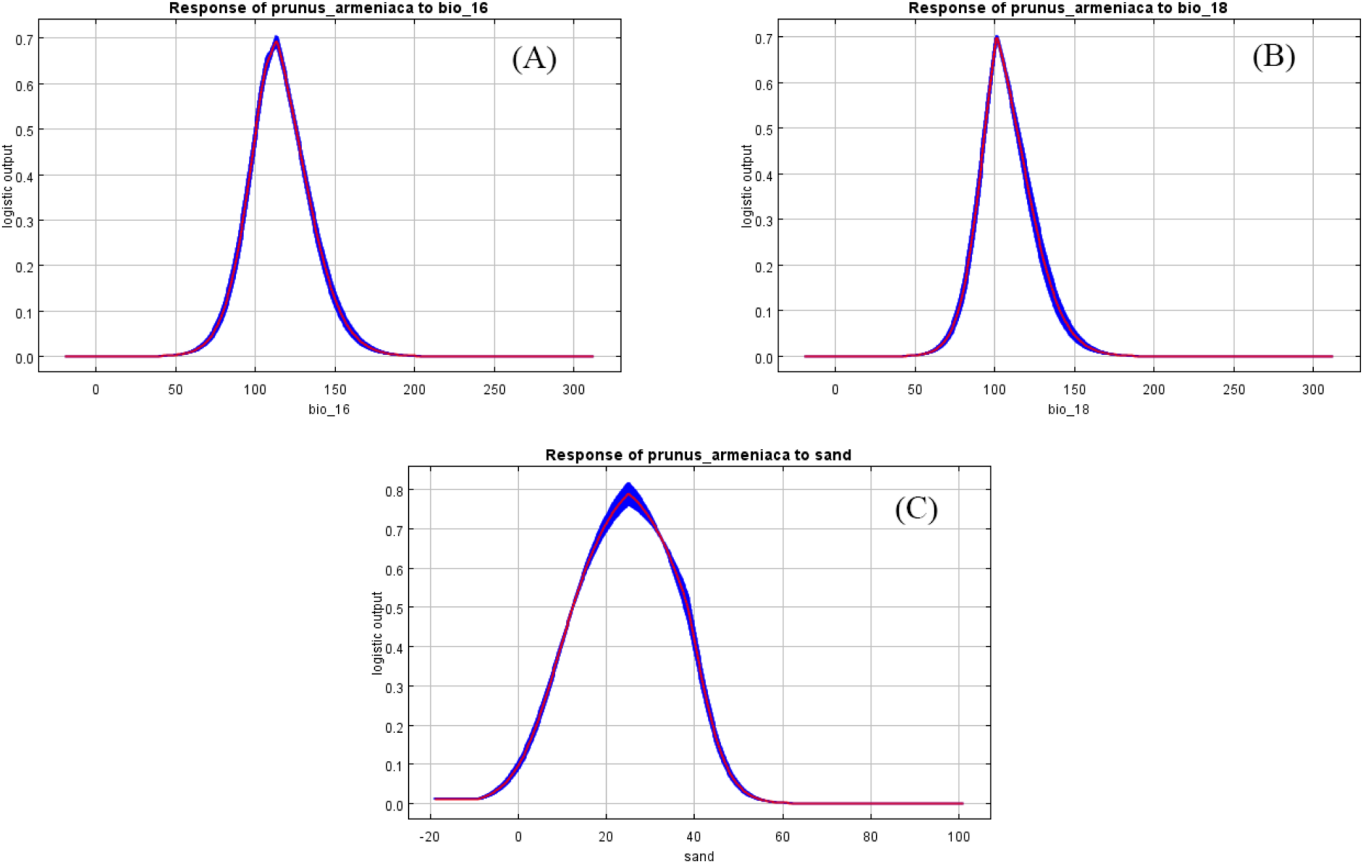
Response curves of *Prunus armeniaca* to dominant environmental factors. A, Precipitation of wettest quarter (Bio 16); B, Precipitation of warmest quarter (Bio 18); C, Sand content.

The MaxEnt model was employed to predict the potential habitat of wild apricot ( *P. armeniaca* ). Results indicated an area under the curve (AUC) of 0.987 (Figure 5), exceeding the 0.9 threshold, which demonstrated high predictive accuracy and reliable outcomes. Further analysis using the Jackknife method identified dominant environmental factors: precipitation of the wettest quarter (Bio 16), precipitation of the warmest quarter (Bio 18), and sand content (Sand) as key variables influencing wild apricot distribution (Figure 6). Based on these factors, response curves for environmental variables were plotted. Using a presence probability threshold of 0.5 as the suitability threshold, the optimal environmental ranges for wild apricot survival were determined as follows: precipitation of the wettest quarter 100–135 mm, precipitation of the warmest quarter 90–120 mm, and sand content 6%–38% (Figure 7).

### 3.5 Results of Ecological Niche Modeling

**FIGURE 8:**
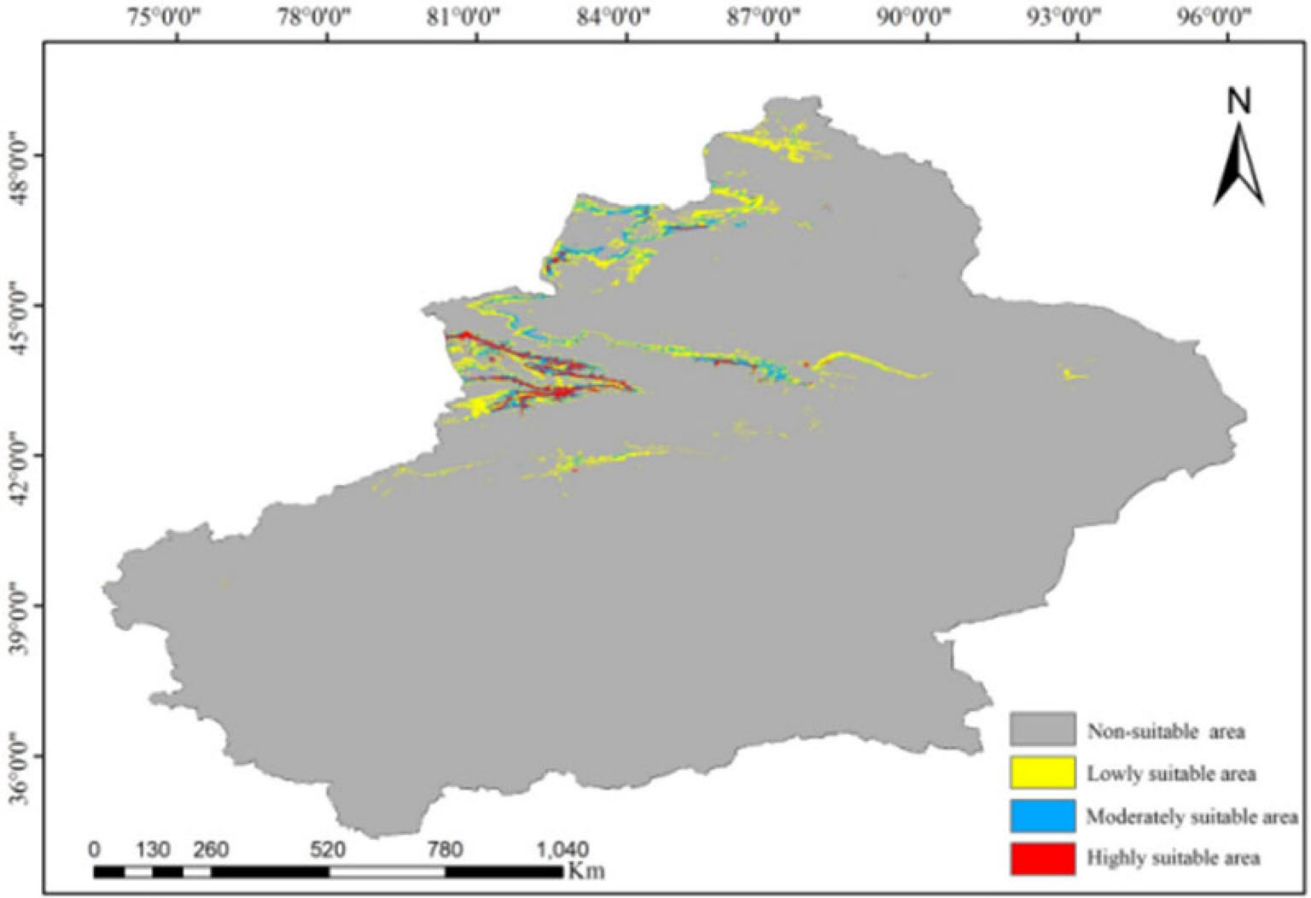
Current potential habitat areas of *Prunus armeniaca* and its related species.

**FIGURE 9:**
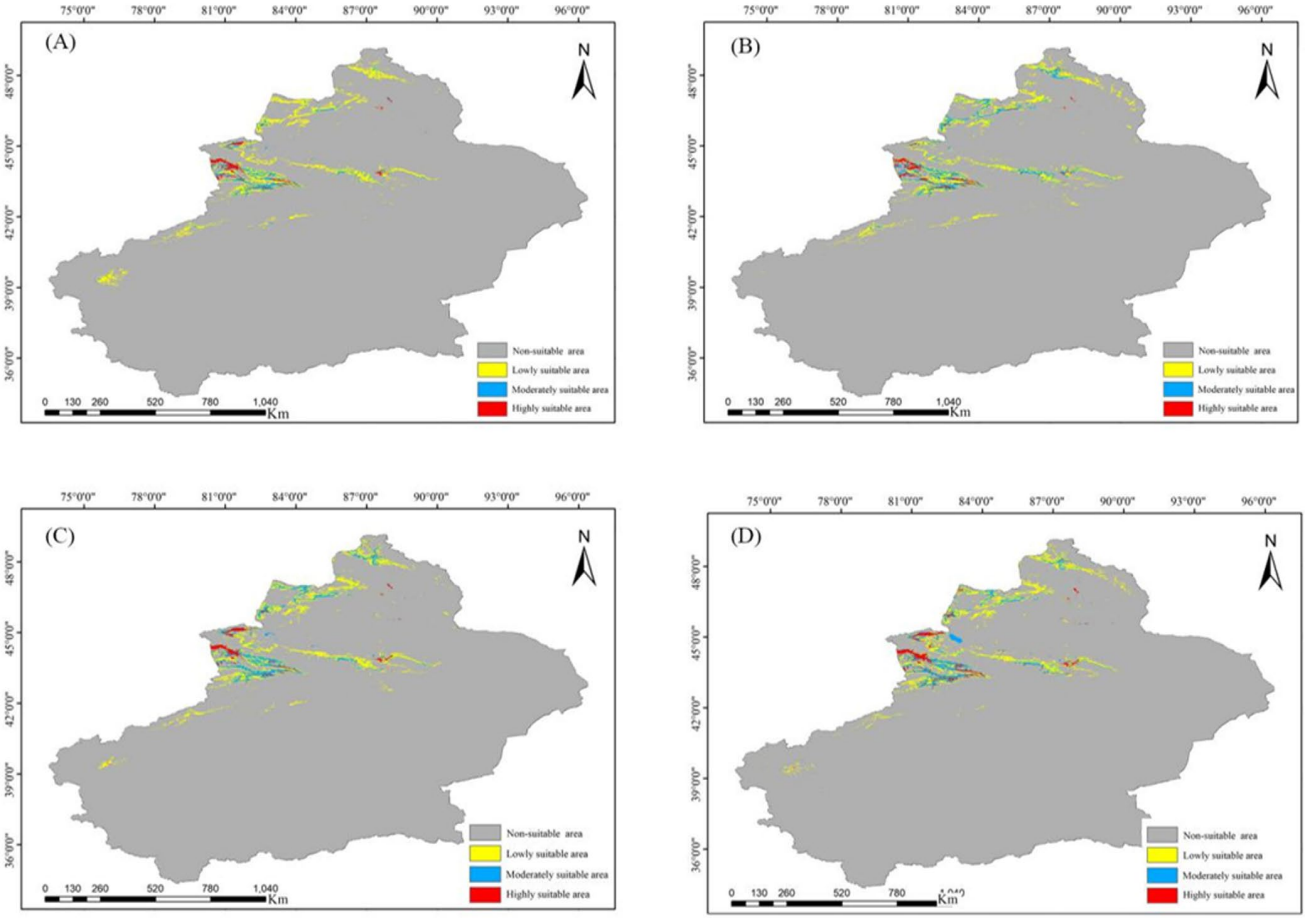
Future habitat regions of *Prunus armeniaca* in the world. (A) Map of *P. armeniaca* suitable areas under 2060‐SSP126 emission conditions; (B) Map of *P. armeniaca* suitable areas under 2080‐SSP126 emission conditions; (C) Map of *P. armeniaca* suitable areas under 2060S‐SSP585 emission conditions; (D) Map of *P. armeniaca* suitable areas under 2080S‐SSP585 emission conditions.

Simulation results indicated that the total suitable habitat area for wild apricot currently spanned approximately 75.13 × 10^3^ km^2^, comprising high, medium, and low suitability zones of 9.42 × 10^3^ km^2^, 22.01 × 10^3^ km^2^, and 43.70 × 10^3^ km^2^, respectively. High‐suitability areas were primarily concentrated in Ili Kazakh Autonomous Prefecture, Tacheng Prefecture, Shihezi City, and Urumqi City (Figure 8, Table S4). Future projections based on the BCC‐CSM2‐MR climate model and MaxEnt model indicate: under the SSP126 pathway, total suitable habitat area decreased in the 2060s but reversed to growth in the 2080s; under the SSP585 pathway, the total suitable habitat area showed an increasing trend in both the 2060s and 2080s (Figure 9). In the SSP585 scenario by the 2080s, the total suitable habitat area reached its maximum (84.78 × 10^3^ km^2^), expanding by 9.65 × 10^3^ km^2^ compared to the present, while the area of highly suitable habitat peaked at 10.30 × 10^3^ km^2^. Conversely, under the SSP126 scenario in the 2080s, the area of highly suitable habitat declined to its lowest point (6.16 × 10^3^ km^2^), representing a reduction of 3.26 × 10^3^ km^2^ compared to current levels.

## 4 Discussion

In interpreting the phylogeographic patterns of wild apricot, the resolution limits of the marker system used in this study should be explicitly acknowledged. Our analyses were based on two cpDNA intergenic regions (*rpl32‐trnL* and *ndhC‐trnV*) and one single‐copy nuclear locus (*DXH*), which provide complementary but limited genomic representation. This combined framework is suitable for detecting broad lineage structure and for contrasting maternal (seed‐mediated) and biparental (seed‐ and pollen‐mediated) historical signals. However, it does not fully resolve fine‐scale, genome‐wide differentiation. In particular, inferences from a single nuclear locus may be influenced by locus‐specific stochastic processes (e.g., incomplete lineage sorting) and therefore may not fully reflect genome‐wide demographic history. Accordingly, our conclusions are framed at the level of major demographic trends and relative differences between inheritance systems, rather than as definitive genome‐wide reconstructions. Within this scope, the concordant and contrasting signals recovered from cpDNA and *DXH* provide robust evidence for reconstructing key aspects of the population history of *P. armeniaca* in Xinjiang. Future whole‐genome resequencing (WGS) based on high‐density SNP data will be required to test these inferences at higher resolution, particularly for recent divergence, admixture dynamics, and local adaptation.

Genetic diversity constitutes a vital component of biological diversity, serving as the foundation for species adaptation to environmental changes, maintenance of ecosystem stability, and advancement of biological evolution. It holds critical significance for species survival and evolution (Jeon et al. 2024; Khade et al. 2025). Based on an SSR analysis of three populations, a previous study reported an average expected heterozygosity (He) of ≈0.287 (I ≈0.458) and an *F*st of ≈0.16, indicating that approximately 84% of the genetic variation originated within populations (He et al. 2007). Simultaneously, phenotypic analyses revealed that the coefficient of variation (CV) for most traits ranged from ≈10.4% to 45.6%, and that populations exhibited geographical clustering in phenotypic trait space (Liu et al. 2015). Collectively, these findings supported an overarching pattern, previously termed “high intrapopulation diversity coupled with moderate interpopulation differentiation.” This study further revealed through cpDNA analysis that wild apricot exhibited moderate diversity at the maternal genetic level (*H*d = 0.646), with five distinct haplotypes identified. Its lineage pattern featured a shared core of h4, including the XHG‐specific h1 and h5 concentrated in the Huocheng County population, forming an overall star‐like distribution pattern (Slatkin and Hudson 1991). Such phylogenetic patterns were generally interpreted as genetic evidence of rapid population expansion from one or more refugia following historical population contractions, such as those induced by Quaternary glaciations (Qiu et al. 2011). Significant differences in cpDNA genetic composition were observed among populations. The PLQG population exhibited haplotype fixation (*H*d = 0), indicating it had undergone a severe genetic bottleneck. In contrast, the XXG and XHG populations maintained high haplotype diversity, suggesting these regions might serve as important maternal genetic diversity conservation hubs for wild apricots in the Ili River Valley. These areas should be prioritized in subsequent conservation strategies. At the *DXH* locus, the genetic diversity of wild apricot (*H*d = 0.902) was significantly higher than previously reported values for several closely related Rosaceae species, including wild cherry (*H*d = 0.562), cherry plum (*H*d = 0.559), and Chinese cherry (*H*d = 0.553) (Chen, Wang, et al. 2012; Chen, Xing, et al. 2012; He et al. 2014; Gao et al. 2024). This result further confirmed the broad genetic base of wild apricot from the perspective of biparental inheritance. The high diversity was consistent with its diploid inheritance, large effective population size, and continuous input of genetic variation through gene flow mediated by pollen and seed dispersal, which aligned with expectations from population genetics theory (Fichant et al. 2023; Browne et al. 2018). At the population level, MZG and YLGD exhibited the highest haplotype and nucleotide diversity, identifying them as key hotspots for nuclear genetic resources of wild apricot. Although the PLQG population retained six nuclear gene haplotypes, its nucleotide diversity (*P*i) remained markedly low. This phenomenon reflects a difference between genetic systems: cpDNA was spread exclusively via maternal seeds, rendering it more susceptible to haplotype fixation during historical bottlenecks; in contrast, nuclear genes were dispersed by both pollen and seeds, which allows exogenous pollen flow to introduce new alleles and partially restore genotypic diversity. Nevertheless, under prolonged constraints of small population size and geographical isolation, genetic drift continuously eliminated low‐frequency alleles. This process diminished the overall genetic variation within the PLQG population, potentially constraining its evolutionary potential (Nagano et al. 2022). Notably, similar star‐like haplotype network structures and widely distributed core haplotypes were observed not only in wild apricot but also in various temperate woody plants across the Ili River Valley and Central Asia, such as *Malus sieversii* (Volk et al. 2012), *Juglans regia* (Zhang, Cao, et al. 2019; Zhang, Wei, et al. 2019), *Ulmus pumila* (Fu et al. 2017), and *Quercus mongolica* (Yang et al. 2016). These studies consistently interpreted such structures as signals of rapid post‐glacial expansion, inferring refuge locations in the Tianshan Mountains or the Ili River Valley and adjacent valleys. The cpDNA structure of wild apricot observed in this study exhibited high similarity to those of the aforementioned species, with overlapping ecogeographical distributions. Consequently, our findings provided cross‐species comparative support for the “postglacial expansion” hypothesis at the regional scale. Comparatively, it was evident that the genetic structures revealed by cpDNA and SCN exhibited distinct differences, primarily stemming from their differing genetic mechanisms and gene flow mediators (Browne et al. 2018). cpDNA was typically maternally inherited, primarily propagated via seeds, and exhibits limited dispersal capacity. Consequently, it more readily preserves genetic signatures of historical bottlenecks, refugial isolation, and subsequent expansions (Guo et al. 2023). In contrast, nuclear genes were inherited biparentally. Gene flow occurs via both seeds and, more significantly, pollen dispersal, which was extensive and frequent. This facilitates the maintenance of genetic homogeneity across larger spatial scales, enhances diversity levels, and reduces interpopulation differentiation (Wessinger 2021). The IBD analysis further corroborated this conclusion. It revealed a significant positive correlation between cpDNA genetic distance and geographical distance (Mantel's *r* = 0.238, *p* = 0.045), whereas nuclear genes exhibited only a weak, nonsignificant positive correlation (*r* = 0.205, *p* = 0.073). This pattern reflected the consequences of restricted maternal gene flow and limited seed dispersal, which caused cpDNA to exhibit a stronger spatial genetic structure than nuclear genes. This phenomenon arises from fundamental differences in genetic transmission. The chloroplast genome is almost exclusively maternally inherited and disseminated via seeds, a process constrained by topography, vegetation, and animal dispersers. In contrast, nuclear genes can achieve long‐distance gene flow via pollen, which dilutes spatial genetic differentiation over broader ranges (Corriveau and Coleman 1988; Mogenson 1996). Furthermore, the complex mountainous and valley topography of the Ili region in Xinjiang likely amplified this pattern. Natural barriers between distant populations not only reduced the probability of seed dispersal but also prolonged periods of genetic isolation, allowing maternal markers to accumulate differences with geographical distance. Conversely, wind‐ or insect‐mediated pollen dispersal partially overcame these topographical constraints, maintaining greater connectivity for nuclear gene flow (Levin et al. 2003). Therefore, SCN exhibited higher *H*d values, reflecting the stronger shaping effect of contemporary or recent gene flow on genetic structure (Guiller et al. 2023). The inconsistency between cpDNA and SCN revealed the complex history of natural selection, genetic drift, and their interactions with gene flow experienced by wild apricots under different genetic systems, and provided a theoretical basis for the future protection and genetic management of their germplasm resources.

Genetic differentiation reflects variations in gene frequencies and genetic structure among different populations or groups (Zhou et al. 2025). In this study, the integration of cpDNA and SCN analyses revealed complex genetic patterns and evolutionary history in wild apricot populations. Genetic structure analysis indicated that genetic variation in wild apricot is primarily maintained within populations. Moderate genetic differentiation among populations (*F*st = 0.150–0.206) suggested that geographical distance plays a pivotal role in shaping the distribution of genetic diversity (Lowe and Allendorf 2010). Notably, cpDNA and SCN markers revealed seemingly contradictory demographic histories. The cpDNA results supported a historical population contraction (bottleneck effect) in the maternal lineage, potentially linked to past climatic upheavals or habitat fragmentation. It should be further noted that the expansion and contraction signals reflected in the cpDNA data may correspond to population events operating at different temporal scales. Neutrality test results did not support a significant overall expansion and instead indicated that the maternal lineage may have experienced population shrinkage or a bottleneck during a relatively recent period. In contrast, the estimated expansion time of haplotype H4 (approximately 19.6 ka), based on the mismatch distribution parameter τ, points to an earlier expansion event, which may be associated with improved climatic conditions and habitat expansion following the end of the Last Glacial Maximum (LGM) (Rogers and Harpending 1992). This pattern suggests that the maternal lineage of wild apricot may have undergone a multi‐stage demographic history, characterized by a post‐glacial expansion followed by a more recent contraction driven by climatic fluctuations, habitat fragmentation, or other environmental pressures, thereby masking earlier expansion signals in the overall cpDNA dataset (G. Hewitt 2000, 2004).

In contrast, the *DXH* data—particularly the significantly negative Tajima's *D* value and unimodal mismatch distribution observed in the Yining County population—collectively indicated a recent population expansion. This discrepancy between chloroplast and nuclear DNA signals suggests that cpDNA records more ancient contraction events, whereas the nuclear genome, characterized by a larger effective population size and slower evolutionary rate, retains a stronger signature of recent expansion (Jeon et al. 2024). In the nuclear gene analyses, the population from Yining County exhibited the clearest signal of recent expansion (Tajima's *D* was significantly negative, and the mismatch distribution was unimodal), which was stronger than that observed in the other populations, indicating heterogeneity in recent demographic dynamics among regions. Yining County is located in the core area of the Ili River Valley, which is characterized by relatively mild climatic conditions, high availability of water and thermal resources, and continuous suitable habitats. These factors may have provided favorable conditions for the rapid recovery and expansion of wild apricot following recent climatic fluctuations. In addition, the Ili River Valley has long been a region with relatively intensive human activities. Agricultural practices and semi‐domestication management may have promoted the survival and dispersal of wild apricot individuals, thereby amplifying the genetic signals of recent expansion at the nuclear gene level. In contrast, the remaining populations may have been more strongly affected by habitat fragmentation or different forms of human disturbance, resulting in weaker expansion signals or signals that were partially offset by subsequent population contractions. It should be noted that the above interpretations are based on integrated inferences from genetic evidence and regional background information, and they require further validation through the incorporation of fine‐scale ecological variables, land‐use history, and genome‐wide data in future studies. In summary, wild apricot demonstrates a strong capacity for recovery and expansion at the nuclear genomic level following an ancient bottleneck. These findings not only enhance our understanding of the species' population history but also provide a genetic basis for assessing its adaptive potential and evolutionary resilience under future environmental change (Najafipour et al. 2023).

ENMs serve as valuable tools for predicting species distributions and their potential responses to future environmental changes (Zhang et al. 2025). This study employed MaxEnt modeling and ArcGIS v. 10.8 to simulate the ecological niche of wild apricot, revealing migration patterns and variation trends in its suitable habitats under different climatic conditions. Currently, wild apricot is primarily distributed across Ili Kazakh Autonomous Prefecture, Bortala Mongol Autonomous Prefecture, and Tacheng Prefecture, with highly suitable habitats concentrated in the Ili and Tacheng regions. It should be noted that the sampling sites in this study were primarily situated in areas with accessible transportation, and the model failed to fully capture ecological processes—such as the barrier effect of the Tianshan Mountains. Therefore, the MaxEnt predictions primarily reflected potential suitable habitats rather than actual distribution ranges. By integrating 19 climatic factors, 3 topographic factors, and 8 soil factors, this study identified the dominant environmental factors affecting the suitable habitats of wild apricot and their thresholds: precipitation of wettest quarter (Bio 16) ranging from 100 to 135 mm, precipitation of warmest quarter (Bio 18) ranging from 90 to 120 mm, and sand content ranging from 6% to 38%. Simulations of future scenarios indicated a slight expansion in the total area of suitable habitats for wild apricot, along with potential for northward migration. Although the model predicted habitat expansion, actual population growth might be constrained by both anthropogenic and natural factors—including grazing, logging, and pest and disease pressures (Qiao et al. 2024; Frans and Liu 2024). Therefore, it is recommended to strengthen field surveys of wild apricot to clarify its actual distribution range, advance the collection of germplasm resources, and conduct research on introduction and propagation, thereby providing support for the scientific conservation and sustainable utilization of wild apricot.

## 5 Conclusion

Based on phylogeographic analyses of cpDNA (*rpl32‐trnL* and *ndhC‐trnV*) and SCN (*DXH*), this study indicated that Xinjiang wild apricot populations exhibited high genetic diversity, with most variation originating within populations. In addition, genetic differentiation existed among populations, forming a distinct phylogeographic structure. The analysis of SCN data generally supported the hypothesis that wild apricot had experienced significant population expansion events in recent evolutionary history. Specifically, the expansion signal in the Yining population was the most pronounced (Tajima's *D* was significantly negative), indicating that this historical expansion event left a significant mark in its nuclear genome. Combined with MaxEnt ecological niche model predictions, dominant environmental factors influencing wild apricot distribution and their thresholds were identified, indicating that contemporary highly suitable areas were primarily concentrated in Ili Kazakh Autonomous Prefecture, Tacheng Prefecture, and the northern slopes of the Tianshan Mountains. Further MaxEnt model projections suggested that under future climate change, the potential suitable habitat range for wild apricot will generally expand. These findings provide a crucial scientific basis for deepening understanding of wild apricot's evolutionary history, responding to climate change, and formulating effective germplasm conservation strategies.

## Author Contributions

**Mingyu Li:** conceptualization (equal), data curation (equal), formal analysis (equal), funding acquisition (equal), investigation (equal), methodology (equal), project administration (equal), resources (equal), software (equal), supervision (equal), validation (equal), visualization (equal), writing – original draft (equal), writing – review and editing (equal). **Xiaolan Wu:** conceptualization (equal), data curation (equal), formal analysis (equal), funding acquisition (equal), investigation (equal), methodology (equal), project administration (equal), resources (equal), software (equal), supervision (equal), validation (equal), visualization (equal), writing – original draft (equal), writing – review and editing (equal). **Mengfan Cui:** software (equal). **Tao Hu:** data curation (equal). **Chenyang Ma:** formal analysis (equal). **Chen Yuan:** formal analysis (equal). **Chenxi Liu:** formal analysis (equal). **Deyin Cao:** formal analysis (equal). **Wenwen Li:** conceptualization (equal), data curation (equal), formal analysis (equal), funding acquisition (equal), investigation (equal), methodology (equal), project administration (equal), resources (equal), software (equal), supervision (equal), validation (equal), visualization (equal), writing – original draft (equal), writing – review and editing (equal). **Kai Jia:** conceptualization (equal), data curation (equal), formal analysis (equal), funding acquisition (equal), investigation (equal), methodology (equal), project administration (equal), resources (equal), software (equal), supervision (equal), validation (equal), visualization (equal), writing – original draft (equal), writing – review and editing (equal).

## Funding

This work was supported by the “Tianshan Talents” Youth Support Program of the Xinjiang Uygur Autonomous Region Association for Science and Technology (Grant No. 2023TSYCQNTJ0038) and the National Natural Science Foundation China (Grant Nos. 32200183 and 32560058).

## Conflicts of Interest

The authors declare no conflicts of interest.

## Supporting information


**Tables S1–S4:** ece373206‐sup‐0001‐Tables.pdf.

## Data Availability

The haplotype sequences generated in this study are openly available in GenBank (https://www.ncbi.nlm.nih.gov/genbank/) under BioProject accession numbers PRJNA1348164 and PRJNA1347998.

## References

[ece373206-bib-0001] Avise, J. C. , J. Arnold , R. M. Ball , et al. 1987. “Intraspecific Phylogeography: The Mitochondrial DNA Bridge Between Population Genetics and Systematics.” Annual Review of Ecology and Systematics 18, no. 1: 489–522. 10.1146/annurev.es.18.110187.002421.

[ece373206-bib-0003] Browne, L. , K. Ottewell , V. L. Sork , and J. Karubian . 2018. “The Relative Contributions of Seed and Pollen Dispersal to Gene Flow and Genetic Diversity in Seedlings of a Tropical Palm.” Molecular Ecology 27, no. 15: 3159–3173. 10.1111/mec.14768. 29924880

[ece373206-bib-0004] Chang, J. G. , L. T. Yan , S. L. Lin , et al. 2021. “Predicting the Potential Global Distribution of *Ageratina adenophora* Under Current and Future Climate Change Scenarios.” Ecology and Evolution 11, no. 17: 12092–12113. 10.1002/ece3.7974. 34522363 PMC8427655

[ece373206-bib-0005] Chaurasia, A. N. , R. M. Parmar , M. G. Dave , and N. S. R. Krishnayya . 2024. “Integrating Field‐and Remote Sensing Data to Perceive Species Heterogeneity Across a Climate Gradient.” Scientific Reports 14, no. 1: 42. 10.1038/s41598-023-50812-y. 38167992 PMC10761838

[ece373206-bib-0006] Chen, J. M. , S. Y. Zhao , Y. Y. Liao , A. W. Gichira , R. W. Gituru , and Q. F. Wang . 2015. “Chloroplast DNA Phylogeographic Analysis Reveals Significant Spatial Genetic Structure of the Relictual Tree *Davidia involucrata* (Davidiaceae).” Conservation Genetics 16, no. 3: 583–593.

[ece373206-bib-0007] Chen, S. , Y. Xing , T. Su , Z. Zhou , E. D. L. Dilcher , and D. E. Soltis . 2012. “Phylogeographic Analysis Reveals Significant Spatial Genetic Structure of *Incarvillea sinensis* as a Product of Mountain Building.” BMC Plant Biology 12, no. 1: 58. 10.1186/1471-2229-12-58. 22546007 PMC3447706

[ece373206-bib-0008] Chen, T. , X. Wang , H. Luo , W. Chun‐Tao , Z. Jia‐Zhi , and L. Ming‐Min . 2012. “Analysis of Chloroplast DNA *trnQ‐rps16* Sequence Variation and Genetic Structure in Nine Wild Chinese Cherry Populations.” Genetics 34, no. 11: 1491–1499. 10.3724/SP.J.1005.2012.01475. 23208145

[ece373206-bib-0009] Corriveau, J. L. , and A. W. Coleman . 1988. “Rapid Screening Method to Detect Potential Biparental Inheritance of Plastid DNA and Results for Over 200 Angiosperm Species.” American Journal of Botany 75, no. 10: 1443–1458. 10.2307/2444695.

[ece373206-bib-0010] Dormann, C. F. , J. Elith , S. Bacher , et al. 2013. “Collinearity: A Review of Methods to Deal With It and a Simulation Study Evaluating Their Performance.” Ecography 36, no. 1: 27–46. 10.1111/j.1600-0587.2012.07348.x.

[ece373206-bib-0011] Feng, J. , X. Dan , Y. Cui , et al. 2024. “Integrating Evolutionary Genomics of Forest Trees to Inform Future Tree Breeding Amid Rapid Climate Change.” Plant Communications 5, no. 10: 101144. 10.1016/j.xplc.2024.101044. PMC11573912 39095989

[ece373206-bib-0012] Fichant, T. , A. Ledent , F. Collart , and A. Vanderpoorten . 2023. “Dispersal Capacities of Pollen, Seeds and Spores: Insights From Comparative Analyses of Spatial Genetic Structures in Bryophytes and Spermatophytes.” Frontiers in Plant Science 14: 1289240. 10.3389/fpls.2023.1289240. 37965033 PMC10642818

[ece373206-bib-0013] Fick, S. E. , and R. J. Hijmans . 2017. “WorldClim 2: New 1‐km Spatial Resolution Climate Surfaces for Global Land Areas.” International Journal of Climatology 37, no. 12: 4302–4315. 10.1002/joc.5086.

[ece373206-bib-0014] Frans, V. F. , and J. Liu . 2024. “Gaps and Opportunities in Modeling Human Influence on Species Distributions in the Anthropocene.” Nature Ecology & Evolution 8, no. 7: 1365–1377. 10.1038/s41559-024-02435-3. 38867092 PMC11239511

[ece373206-bib-0015] Fu, C. , D. Jiang , M. Sun , et al. 2017. “Phylogeography of *Ulmus pumila* (Ulmaceae) in Arid and Semi‐Arid Regions of Northern China: Insights Into Species Response to Climate Change.” Molecular Phylogenetics and Evolution 109: 354–363. 10.1016/j.indcrop.2025.121452.

[ece373206-bib-0016] Gao, S. , X. Chen , Z. Peng , et al. 2024. “Species Differentiation of *Prunus serrulata* and *Prunus xueluoensis* Based on Combined Analysis of SSR and cpDNA Markers.” Forests 15, no. 11: 1927. 10.3390/f15111927.

[ece373206-bib-0017] Groppi, A. , S. Liu , A. Cornille , et al. 2021. “Population Genomics of Apricots Unravels Domestication History and Adaptive Events.” Nature Communications 12, no. 1: 3956. 10.1038/s41467-021-24283-6. PMC8233370 34172741

[ece373206-bib-0018] Guiller, A. , G. Decocq , T. Kichey , et al. 2023. “Spatial Genetic Structure of Two Forest Plant Metapopulations in Dynamic Agricultural Landscapes.” Landscape and Urban Planning 231: 104648. 10.1016/j.landurbplan.2022.104648.

[ece373206-bib-0020] Guo, C. , Y. He , X. Zeng , et al. 2023. “Chloroplast DNA Reveals Genetic Population Structure in *Sinomenium acutum* in Subtropical China.” Chinese Herbal Medicines 15, no. 2: 278–283. 10.1016/j.chmed.2022.11.003. 37265762 PMC10230624

[ece373206-bib-0021] He, S. , F. Pan , C. Jiang , J. Feng , C. Zhao , and J. J. Wang . 2025. “Unraveling the Genetic Diversity and Phylogeography of the ‘King of Vitamin C’ Fruit (*Rosa roxburghii* Trattinnick) in Chinese Southwest.” Ecology and Evolution 15, no. 5: e71369. 10.1002/ece3.71369. 40342695 PMC12058451

[ece373206-bib-0022] He, T. M. , X. S. Chen , H. Tian‐Ming , et al. 2007. “Using SSR Markers to Determine the Population Genetic Structure of Wild Apricot (*Prunus armeniaca* L.) in the Ily Valley of West China.” Genetic Resources and Crop Evolution 54, no. 3: 563–572. 10.1007/s10722-006-0013-5.

[ece373206-bib-0023] He, W. , J. Zhang , Z. I. Huang , et al. 2014. “Analysis of Genetic Diversity and Population Structure of Cultivated Chinese Cherry Based on ITS Sequences.” Acta Botanica Sinica 34, no. 3: 463–472. 10.1007/s11105-015-0934-2.

[ece373206-bib-0024] Hewitt, G. 2000. “The Genetic Legacy of the Quaternary Ice Ages.” Nature 405, no. 6789: 907–913. 10.1038/35016000. 10879524

[ece373206-bib-0025] Hewitt, G. M. 2004. “Genetic Consequences of Climatic Oscillations in the Quaternary.” Philosophical Transactions of the Royal Society of London. Series B: Biological Sciences 359, no. 1442: 183–195. 10.1098/rstb.2003.1388. 15101575 PMC1693318

[ece373206-bib-0026] Hu, X. , S. Du , Y. Han , et al. 2021. “Genome‐Scale Mining of Single‐Copy Nuclear Gene Markers for *Ziziphus jujuba* Var. Spinosa and Implications for Genetic Studies.” Pakistan Journal of Botany 53, no. 4: 60. 10.1186/s12862-015-0416-z.

[ece373206-bib-0027] Jeon, J. Y. , Y. Shin , A. J. Mularo , X. Feng , and J. A. DeWoody . 2024. “The Integration of Whole‐Genome Resequencing and Ecological Niche Modeling to Conserve Profiles of Local Adaptation.” Diversity and Distributions 30, no. 6: e13847. 10.1111/ddi.13847.

[ece373206-bib-0028] Khade, Y. , D. P. Mainkar , D. A. Chandanshive , et al. 2025. “Harnessing Chloroplast SSRs to Decipher Genetic Diversity in Underutilized *Allium* Species.” Frontiers in Plant Science 16: 1645145. 10.3389/fpls.2025.1645145. 41031300 PMC12477137

[ece373206-bib-0029] Levin, S. A. , H. C. Muller‐Landau , R. Nathan , H. C. Muller‐Landau , R. Nathan , and J. Chave . 2003. “The Ecology and Evolution of Seed Dispersal: A Theoretical Perspective.” Annual Review of Ecology, Evolution, and Systematics 34, no. 1: 575–604. 10.1146/annurev.ecolsys.34.011802.132428.

[ece373206-bib-0030] Li, J. , Y. Du , L. Xie , X. Jin , Z. Zhang , and M. Yang . 2023. “Comparative Plastome Genomics and Phylogenetic Relationships of the Genus Trollius.” Frontiers in Plant Science 14: 1293091. 10.3389/fpls.2023.1293091. 38046610 PMC10690957

[ece373206-bib-0031] Li, J. , Y. Zhang , M. Ruhsam , et al. 2022. “Seeing Through the Hedge: Phylogenomics of *Thuja* (Cupressaceae) Reveals Prominent Incomplete Lineage Sorting and Ancient Introgression for Tertiary Relict Flora.” Cladistics 38, no. 2: 187–203. 10.1111/cla.12491. 34551153

[ece373206-bib-0032] Li, W. W. , L. Q. Liu , Y. N. Wang , et al. 2020. “Genetic Diversity, Population Structure, and Relationships of Apricot (*Prunus*) Based on Restriction Site‐Associated DNA Sequencing.” Horticulture Research 7, no. 1: 69. 10.1038/s41438-020-0284-6. 32377359 PMC7192913

[ece373206-bib-0033] Li, W. W. , L. Q. Liu , Q. P. Zhang , W. Q. Zhou , G. Q. Fan , and K. Liao . 2021. “Phylogeography of *Prunus armeniaca* L. Revealed by Chloroplast DNA and Nuclear Ribosomal Sequences.” Scientific Reports 11: 13623. 10.1038/s41598-021-93050-w. 34211010 PMC8249649

[ece373206-bib-0034] Li, Y. , L. Wang , X. Zhang , et al. 2022. “Extensive Sharing of Chloroplast Haplotypes Among East Asian *Cerris oaks*: The Imprints of Shared Ancestral Polymorphism and Introgression.” Ecology and Evolution 12, no. 8: e9142. 10.1002/ece3.9142. 35923946 PMC9339761

[ece373206-bib-0035] Liu, J. , K. Liao , H. Liu , et al. 2015. “Study on Phenotypic Trait Diversity of Wild Apricot Germplasm Resources in Xinjiang.” Acta Botanica Sinica 35, no. 5: 1021–1030. 10.7606/j.issn.1000-4025.2015.05.1021.

[ece373206-bib-0036] Liu, S. , J. Gao , B. Xiao , et al. 2025. “Genetic Differentiation and Historical Dynamics of the Endemic Species *Rheum pumilum* on the Qinghai‐Tibetan Plateau Inferred From Phylogeography Implications.” BMC Plant Biology 25, no. 1: 162. 10.1186/s12870-025-06164-y. 39915721 PMC11803965

[ece373206-bib-0037] Lowe, W. H. , and F. W. Allendorf . 2010. “What Can Genetics Tell Us About Population Connectivity?” Molecular Ecology 19, no. 15: 3038–3051. 10.1111/j.1365-294X.2010.04688.x. 20618697

[ece373206-bib-0038] Médail, F. , and K. Diadema . 2009. “Glacial Refugia Influence Plant Diversity Patterns in the Mediterranean Basin.” Journal of Biogeography 36, no. 7: 1333–1345. 10.1111/j.1365-2699.2008.02051.x.

[ece373206-bib-0039] Mogenson, H. L. 1996. “The Hows and Whys of Cytoplasmic Inheritance in Seeds Plants.” American Journal of Botany 83: 383–404. 10.1002/j.1537-2197.1996.tb12718.x.

[ece373206-bib-0040] Moraira, H. S. , D. Vitales , N. Nualart , et al. 2020. “Global Distribution Patterns and Niche Modeling of the Invasive *Kalanchoe × Houghtonii* (Crassulaceae).” Scientific Reports 10, no. 1: 3143. 10.1038/s41598-020-60079-2. 32081991 PMC7035272

[ece373206-bib-0041] Muscarella, R. , P. J. Galante , M. Soley‐Guardia , et al. 2014. “ENM Eval: An R Package for Conducting Spatially Independent Evaluations and Estimating Optimal Model Complexity for Maxent Ecological Niche Models.” Methods in Ecology and Evolution 5, no. 11: 1198–1205. 10.1111/2041-210X.12261.

[ece373206-bib-0042] Nagano, Y. , H. Tashiro , S. Nishi , N. Hiehata , A. J. Nagano , and S. Fukuda . 2022. “Genetic Diversity of Loquat (*Eriobotrya japonica*) Revealed Using RAD‐Seq SNP Markers.” Scientific Reports 12, no. 1: 10200. 10.1038/s41598-022-14358-9. 35739209 PMC9226044

[ece373206-bib-0043] Najafipour, B. , A. Mirlohi , M. M. Majidi , G. Saeidi , and M. Abtahi . 2023. “Wild Barley Genomic Resources for Drought Adaptability and Quality Improvement.” Journal of Cereal Science 114: 103802. 10.1016/j.jcs.2023.103802.

[ece373206-bib-0044] Qiao, H. , A. T. Peterson , C. E. Myers , Q. Yang , and E. E. Saupe . 2024. “Ecological Niche Conservatism Spurs Diversification in Response to Climate Change.” Nature Ecology & Evolution 8, no. 4: 729–738. 10.1038/s41559-024-02344-5. 38374186 PMC11009114

[ece373206-bib-0045] Qin, A. , B. Liu , Q. Guo , et al. 2017. “Maxent Modeling for Predicting Impacts of Climate Change on the Potential Distribution of *Thuja sutchuenensis* Franch., an Extremely Endangered Conifer From Southwestern China.” Global Ecology and Conservation 10: 139–146. 10.1016/j.gecco.2017.02.004.

[ece373206-bib-0046] Qin, Q. , Y. Dong , J. Chen , et al. 2025. “Comparative Analysis of Chloroplast Genomes Reveals Molecular Evolution and Phylogenetic Relationships Within the *Papilionoideae* of *Fabaceae* .” BMC Plant Biology 25, no. 1: 1–20. 10.1186/s12870-025-06138-0. 39910427 PMC11800526

[ece373206-bib-0047] Qiu, Y. X. , C. X. Fu , and H. P. Comes . 2011. “Plant Molecular Phylogeography in China and Adjacent Regions: Tracing the Genetic Imprints of Quaternary Climate and Environmental Change in the World's Most Diverse Temperate Flora.” Molecular Phylogenetics and Evolution 59, no. 1: 225–244. 10.1016/j.ympev.2011.01.012. 21292014

[ece373206-bib-0048] Rogers, A. R. , and H. Harpending . 1992. “Population Growth Makes Waves in the Distribution of Pairwise Genetic Differences.” Molecular Biology and Evolution 9, no. 3: 552–569. 10.1093/oxfordjournals.molbev.a040727. 1316531

[ece373206-bib-0049] Shi, J. , M. Xia , G. He , et al. 2024. “Predicting *Quercus gilva* Distribution Dynamics and Its Response to Climate Change Induced by GHG Emissions Through MaxEnt Modeling.” Journal of Environmental Management 357: 120841. 10.1016/j.jenvman.2024.120841. 38581898

[ece373206-bib-0050] Slatkin, M. , and R. R. Hudson . 1991. “Pairwise Comparisons of Mitochondrial DNA Sequences in Stable and Exponentially Growing Populations.” Genetics 129, no. 2: 555–562. 10.1093/genetics/129.2.555. 1743491 PMC1204643

[ece373206-bib-0051] Stephens, M. , N. J. Smith , and P. Donnelly . 2001. “A New Statistical Method for Haplotype Reconstruction From Population Data.” American Journal of Human Genetics 68, no. 4: 978–989. 10.1086/319501. 11254454 PMC1275651

[ece373206-bib-0075] Volk, G. M. , C. T. Chao , J. Norelli , et al. 2012. “Genetic Diversity and Structure of *Malus sieversii*, a Wild Progenitor Species of Domesticated Apple, From Central Asia.” Tree Genetics & Genomes 8, no. 2: 261–273. 10.1007/s11295-008-0190-9.

[ece373206-bib-0052] Wang, L. , D. F. Cui , P. J. Lin , et al. 1997. “Subspecies of Wild Apricot in Xinjiang.” Journal of Xinjiang Normal University (Natural Science Edition) 3: 31–36.

[ece373206-bib-0053] Wang, Z. , Y. Zeng , Z. Zhang , et al. 2017. “Phylogeography Study of the Siberian Apricot (*Prunus sibirica* L.) in Northern China Assessed by Chloroplast Microsatellite and DNA Markers.” Frontiers in Plant Science 8: 1989. 10.3389/fpls.2017.01989. 29209348 PMC5702509

[ece373206-bib-0054] Warren, D. L. , and S. N. Seifert . 2011. “Ecological Niche Modeling in Maxent: The Importance of Model Complexity and the Performance of Model Selection Criteria.” Ecological Applications 21, no. 2: 335–342. 10.1890/10-1171.1. 21563566

[ece373206-bib-0055] Wei, B. O. , R. Wang , K. Hou , B. Wei , X. Wang , and W. Wu . 2018. “Predicting the Current and Future Cultivation Regions of *Carthamus tinctorius* L. Using MaxEnt Model Under Climate Change in China.” Global Ecology and Conservation 16: e00477. 10.1016/j.gecco.2018.e00477.

[ece373206-bib-0056] Wessinger, C. A. 2021. “From Pollen Dispersal to Plant Diversification: Genetic Consequences of Pollination Mode.” New Phytologist 229, no. 6: 3125–3132. 10.1016/j.baae.2020.10.007. 33159813

[ece373206-bib-0057] Witharana, E. P. , T. Iwasaki , M. H. San , et al. 2025. “Subfamily Evolution Analysis Using Nuclear and Chloroplast Data From the Same Reads.” Scientific Reports 15, no. 1: 687. 10.1038/s41598-024-83292-9. 39753617 PMC11698846

[ece373206-bib-0058] Wu, M. , Y. Cheng , C. Jiang , M. Zhang , T. Shi , and C. Zhao . 2024. “Phylogeography of *Morella nana*: The Wumeng Mountains as a Natural Geographical Isolation Boundary on the Yunnan‐Guizhou Plateau.” Ecology and Evolution 14, no. 7: e11566. 10.1002/ece3.11566. 38983704 PMC11232048

[ece373206-bib-0059] Xu, T. , R. J. Abbott , R. I. Milne , et al. 2010. “Phylogeography and Allopatric Divergence of Cypress Species (*Cupressus* L.) in the Qinghai‐Tibetan Plateau and Adjacent Regions.” BMC Evolutionary Biology 10, no. 1: 194. 10.1186/1471-2148-10-194. 20569425 PMC3020627

[ece373206-bib-0060] Xue, N. , K. Li , K. Chen , et al. 2024. “Predicting Climate Change Impacts on Distribution and Conservation of Critically Endangered *Picea neoveitchii* Using MaxEnt.” Frontiers in Forests and Global Change 7: 1472857. 10.3389/ffgc.2024.1472857.

[ece373206-bib-0061] Yang, J. , X. Di , X. Meng , L. Feng , Z. Liu , and G. Zhao . 2016. “Phylogeography and Evolution of Two Closely Related Oak Species (Quercus) From North and Northeast China.” Tree Genetics & Genomes 12, no. 5: 89. 10.1007/s11295-016-1044-5.

[ece373206-bib-0062] Ye, J. , F. Yang , J. Hu , et al. 2025. “Improving the Accuracy of Predicted Potential Distributions and Enhancing the Effectiveness of Priority Conservation Areas for Protected Species by Expanding the Target Area.” Diversity and Distributions 31, no. 9: e70087. 10.1111/ddi.70087.

[ece373206-bib-0063] Yi, X. G. , J. Chen , H. Zhu , et al. 2020. “Phylogeography and the Population Genetic Structure of Flowering Cherry *Cerasus serrulata* (Rosaceae) in Subtropical and Temperate China.” Ecology and Evolution 10, no. 20: 11262–11276. 10.1002/ece3.6765. 33144963 PMC7593168

[ece373206-bib-0064] Yousefzadeh, H. , N. Amirchakhmaghi , B. Naseri , F. Shafizadeh , G. Kozlowski , and Ł. Walas . 2022. “The Impact of Climate Change on the Future Geographical Distribution Range of the Endemic Relict Tree *Gleditsia caspica* (Fabaceae) in Hyrcanian Forests.” Ecological Informatics 71: 101773. 10.1016/j.ecoinf.2022.101773.

[ece373206-bib-0065] Zhang, G. , K. Cao , X. Wang , et al. 2019. “Genetic Diversity and Population Structure of Walnut (*Juglans regia*) in China Revealed by cpDNA Markers.” Forests 10, no. 3: 263. 10.1007/s11295-016-1064-1.

[ece373206-bib-0066] Zhang, H. , J. M. Chase , and J. Liao . 2024. “Habitat Amount Modulates Biodiversity Responses to Fragmentation.” Nature Ecology & Evolution 8, no. 8: 1437–1447. 10.1038/s41559-024-02445-1. 38914711

[ece373206-bib-0067] Zhang, J. J. , X. Wei , S. F. Chai , et al. 2019. “Genetic Diversity and Population Structure of *Garcinia paucinervis*, an Endangered Species Using Microsatellite Markers.” Conservation Genetics 20, no. 4: 837–849. 10.1007/s10592-019-01176-2.

[ece373206-bib-0068] Zhang, J. Y. , and Z. Zhang . 2003. Flora of Chinese Fruit Trees. China Forestry Press.

[ece373206-bib-0069] Zhang, W. , X. Wang , S. Shen , et al. 2024. “Analyzing the Distribution Patterns and Dynamic Niche of *Magnolia grandiflora* L. in the United States and China in Response to Climate Change.” Frontiers in Plant Science 15: 1440610. 10.3389/fpls.2024.1440610. 39502915 PMC11534871

[ece373206-bib-0070] Zhang, X. F. , M. M. Nizamani , C. Jiang , F. Z. Fang , and K. K. Zhao . 2024. “Potential Planting Regions of *Pterocarpus santalinus* (Fabaceae) Under Current and Future Climate in China Based on MaxEnt Modeling.” Ecology and Evolution 14, no. 6: e11409. 10.1002/ece3.11409. 38826162 PMC11139971

[ece373206-bib-0071] Zhang, Y. , M. Li , X. Zhang , Z. Qin , P. Wang , and H. Liu . 2025. “Prediction of Potential Suitable Habitats of *Malania oleifera* Under Future Climate Scenarios Based on the MaxEnt Model.” Scientific Reports 15, no. 1: 26422. 10.1038/s41598-025-09800-7. 40691193 PMC12280065

[ece373206-bib-0072] Zhao, Y. J. , G. S. Yin , Y. Z. Pan , B. Tian , and X. Gong . 2021. “Climatic Refugia and Geographical Isolation Contribute to the Speciation and Genetic Divergence in Himalayan‐Hengduan Tree Peonies (*Paeonia delavayi* and *Paeonia ludlowii*).” Frontiers in Genetics 11: 595334. 10.3389/fgene.2020.595334. 33584794 PMC7874331

[ece373206-bib-0073] Zhou, P. , B. Huang , J. Huang , L. A. Xu , Q. Wen , and T. Pyhäjärvi . 2025. “Genetic Differentiation and Associated Climatic Variables Between *Camellia chekiangoleosa* and Its Wild Relatives Revealed by SNP Markers.” Industrial Crops and Products 234: 121594. 10.1016/j.indcrop.2025.121594.

[ece373206-bib-0074] Zhu, X. L. , J. Wang , H. F. Chen , and M. Kang . 2024. “Lineage Differentiation and Genomic Vulnerability in a Relict Tree From Subtropical Forests.” Evolutionary Applications 17, no. 11: e70033. 10.1111/eva.70033. 39494192 PMC11530410

