# Supplementary material for: Phylogeographic and Potential Distribution of Wild Apricot (Prunus armeniaca) in Xinjiang: Insights From Chloroplast/Nuclear DNA and Ecological Niche Modeling
Source: Ecol Evol. 2026 Mar 9;16(3):e73206. doi: 10.1002/ece3.73206 (PMC12971293; doi:10.1002/ece3.73206)
Supplement: Supplementary file 1 — Tables S1–S4: ece373206‐sup‐0001‐Tables.pdf. [file ECE3-16-e73206-s001.pdf]

TABLE S1 Primers and PCR protocols for *P. armeniaca.*

Gene	Primer sequences (5'-3')	Amplification program	Sequence length
rpl32-trnL	F: ATCTCTTTCTACCGGGAGTT<br>R: AGCAACCCGATTAAGTTAGG	94 °C 5 min; 32 cycles: 94 °C 30 s, 52°C 45 s, 72 °C 30 s; 72 °C 10 min.	562 bp
ndhC-trnV	F: CGAAATTGTAACCAAGCATCCC<br>R: GATCTGTTTTACCGAGAAGGTCTA	94 °C 5 min; 32 cycles: 94 °C 30 s, 57 °C 45 s, 72 °C 30 s; 72 °C 10 min.	737 bp
DXH	F: GACGGCAAGTACTACCCGGA<br>R: CTAGCTACAACAGCCACTGC	94 °C 5 min; 35 cycles: 94 °C 30 s, 52 °C 45 s, 72 °C 45 s; 72 °C 10 min.	494 bp

TABLE S2 Parameters of mismatch distribution analysis and neutrality tests for the *P. armeniaca* h4 lineage.

Lineage	τ	θ_0	θ_1	Ramos-Onsins and Rozas's R_2	Raggedness	Time (years)
h4	0.07	9.371	1000	0.2452 (1.00)	0.5425 (1.00)	19600

Note: τ, expansion time parameter; θ_0 and θ_1, represent the effective population size before and after expansion, respectively (where the upper limit is set in the θ_1 simulation). Raggedness: Harpending's raggedness index. The *P*-value was calculated based on 1000 coalescent simulations (P>0.05 indicates consistency with the population expansion model). The expansion time (Time) was calculated according to the formula $t=\tau/2\mu L$, where the mutation rate μ was set to 1.5×10^{-9}, and the sequence length L was 1192 bp.

TABLE S3 Mismatch distribution analysis parameters for *P. armeniaca* populations.

Populations	Xinyuan County population	Gongliu County population	Yining County population	Huocheng County population
**cpDNA**				
Demgraphic expansion				
SSD	0.269	0.332	0.278	0.317
P-value	0	0	0	0.006
Raggedness index	0.462	0.744	0.508	0.614
P-value	0.070	0.810	0	0
Spatial expansion				
SSD	0.160	0.211	0.068	0.220
P-value	0.060	0	0.220	0.010
Raggedness index	0.462	0.744	0.675	0.615
P-value	0.250	0.210	0.615	0.020
**SCN**				
Demgraphic expansion				
SSD	0.064	0.082	0.032	0.043
P-value	0.140	0.060	0.240	0.080
Raggedness index	0.044	0.031	0.080	0.026
P-value	0.360	0.600	0.210	0.520

Spatial expansion				
SSD	0.041	0.055	0.030	0.034
P-value	0.260	0.060	0.280	0.440
Raggedness index	0.044	0.031	0.080	0.258
P-value	0.610	0.720	0.410	0.780

TABLE S4 Changes in potential suitable habitat areas the area (×10^3 km^2) for *P. armeniaca* under future climate scenarios.

**Specie**	**Time**		**Total Suitable area**	**Highly Suitable area**	**Moderately suitable area**	**Lowly Suitable area**
	Contemporary		75.13	9.42	22.01	43.70
	SSP126	2060	70.35	6.29	15.19	48.87
P. armeniaca		2080	81.21	6.16	25.01	50.04
	SSP585	2060	83.12	6.97	26.01	50.14
		2080	84.78	10.30	25.86	48.62
